# Localization and Speciation of Chromium in *Coptis chinensis* Franch. using Synchrotron Radiation X-ray Technology and Laser Ablation ICP-MS

**DOI:** 10.1038/s41598-018-26774-x

**Published:** 2018-06-05

**Authors:** Wenli Huang, Jie Jiao, Mei Ru, Zhenqing Bai, Honglin Yuan, Zhian Bao, Zongsuo Liang

**Affiliations:** 1 College of Life Science, Northwest A&F University, Yangling, China; 2 Institute of Soil and Water Conservation, Chinese Academy of Sciences, Yangling, China; 3 State Key Laboratory of Continental Dynamics, Department of Geology, Northwest University, Xi’an, China; 4 College of Life Sciences, Zhejiang Sci-Tech University, Hangzhou, China

## Abstract

*Coptis chinensis* Franch. is one of the most important medicinal plants globally. However, this species contains relatively high concentrations of chromium (Cr) which potentially detrimental to human health. It is important to understand Cr localization and speciation in order to evaluate its accumulation and transportation mechanisms and minimize Cr transfer to humans. As little previous work in this area has been carried out, we utilized synchrotron radiation microscopic X-ray fluorescence (SR-μXRF) and laser ablation inductively coupled plasma mass spectrometry (LA-ICP-MS) to spatially locate Cr, X-ray absorption near-edge spectroscopy (XANES) to analyze Cr speciation, and inductively coupled plasma mass spectrometry (ICP-MS) to detect Cr subcellular concentration. Micromapping results showed that Cr was distributed predominantly within the vascular cylinder, the periderm and some outer cortex, and the cortex and some vascular bundles in root, rhizome, and petiole, respectively. XANES data showed that Cr(VI) can be reduced to Cr(III) when grown with Cr(VI), and yielded a novel conclusion that this plant contain elemental chromium. ICP-MS data showed that Cr was primarily compartmentalized in cell walls in all tissues. The new insights on Cr accumulation in *C. chinensis* Franch. provide a theoretical basis for the evaluation of Cr in other medicinal plants.

## Introduction

*Coptis chinensis* Franch. is one of the most important medicinal plants in China. This species predominantly occurs in the west of China (Chongqing Municipality, and Hubei and Shaanxi Provinces) and its rhizome (coptidis rhizoma) is commonly used to treat diarrhea, fever, and eczema^1^. To date, more than 30 alkaloid compounds have been isolated from the rhizome of *C. chinensis* Franch^2–4^, including berberine, coptisine, and palmatine; all of these chemical components have been shown to possess antibacterial, antiviral, anti-inflammatory, and antitumor activities-related attributes^1,5^. Because the rhizomes of *C. chinensis* Franch. contain high alkaloids levels, this plant is often referred to as the ‘antibiotic of Chinese medicine’.

Heavy metals levels in medicinal plants have attracted worldwide interest in recent years because these elements normally enter the food chain via uptake by plant and are passed progressively on to the final consumers, leading to a large number health problems^6^. Chromium (Cr)-induced plants and thus food safety has recently gained substantial attention worldwide^7–9^. Cr uptake by vegetables and the accumulation of Cr in the edible parts of plants can lead to numerous consumer health risks^7,10^. In humans, excessive Cr can induce clinical disorders such as the formation of cancers and respiratory, hepatic, renal, cardiovascular, reproductive, and developmental, genotoxic and mutagenic health issues^7^. Cr is one of the most common heavy metals in soils, water, air, sediments, animals, and plants^11^. Cr is generated by a number of natural and anthropogenic activities^7,12^, and significant concentrations of Cr are released into the environment by human endeavors such as leather tanning, metallurgy, electroplating, and textile dyes as well as the production of paints and paper^7,13,14^. Cr is also released into the environment from geological^15^ and other natural sources, such as dust from rocks and volcanic activity^7^.

Although Cr occurs in several oxidation states that range between −2 and +6, its trivalent [Cr(III)] and hexavalent chromate [Cr(VI)] forms are the most common and stable in natural^12^. Of these, Cr(III) is necessary for lipid and sugar metabolism and is an essential trace element for human and animal health^7,16^. Cr, however, does not have any known biological role in plant physiological and biochemical metabolism^17^. Compared with Cr(III), Cr(VI) is more soluble and more toxic^18^. Cr phytotoxicity results in reduced growth and biomass production, degrading pigment status and nutrient balance, and even promotes structural alterations, by inhibiting enzymatic activities and triggering mutagenesis^17,19–21^. However, although plants can uptake both Cr(III) and Cr(VI), the Cr uptake mechanism of plants remains unclear^7^. As it is not an element that is essential for plants, Cr is mostly absorbed by specific carriers for essential ions in plant metabolism^7,11^, while Cr(III) uptake is a passive mechanism and does not require the input of any energy^11^. In contrast, Cr(VI) uptake is active^21^, generally utilizing either phosphate or sulfate transporters because of their structural resemblance to one another^22,23^. Furthermore, Cr(VI) is reported to interfere with the uptake of some essential nutrients (e.g., P, K, Fe, Ca, Mn, and Mg) due to their ionic resemblance^7,24^.

To date, neither *Chinese Pharmacopoeia* (2015) nor *Green Trade Standards of Importing & Exporting Medicinal Plants & Preparations* have set limits on the levels of Cr in medicinal plants. It has been reported that the concentration of this element in plants generally ranges between 0.05 mg kg^−1^ and 1 mg kg^−1^ ^25^. However, we found that the Cr levels between 2.48 mg kg^−1^ and 7.90 mg kg^−1^ in coptidis rhizoma (the rhizome) collected from three locations within Zhenping County in the city of Ankang, Shaanxi Province, China (Supplementary Table S1). These Cr levels are much higher than is generally the case in plants. Similarly, there have been other previous reports^26,27^ showed that Cr concentration was high in coptidis rhizoma in the agricultural environment. Therefore, research is urgently needed on Cr accumulation in *C. chinensis* Franch.

It is important to determine both the localization and speciation characteristics of the heavy metals in plants in order to understand their accumulation and transportation mechanisms^28,29^ as these are regulated by various physiological processes including transport of metals across the plasma membranes of root cells, detoxification, and sequestration of metals in cell walls or vacuoles, xylem loading, and translocation from roots to shoots^30^. Once it is absorbed by roots, Cr is initially retained largely within the roots and later transported throughout the plant by carrier ions^19^. Cr is principally transported through the xylem; and the reduction of Cr(VI) to Cr(III) can occur in the root, within the rhizosphere^31^ or in the aerial parts of plants^21,32^. Several studies have explored the localization and speciation of Cr in a range of vegetables and hyperaccumulators, including subterranean clover (*Trifolium brachycalycium*)^18^, *Lycopersicum esculentum* Mill^33^, *Gynura pseudochina* (L.) DC^34^, *Callitriche cophocarpa* Sendtn^35^, and *Typha angustifolia*^36^. However, the accumulation of this metal in medicinal plants, including *C. chinensis* Franch., remains poorly understood.

Synchrotron radiation microprobe X-ray fluorescence (SR-μXRF) has been widely applied to explore the distribution of elements and has proven a promising tool when employed *in vivo* to study the localization of metals in plants^29^. At the same time, laser ablation inductively coupled plasma mass spectrometry (LA-ICP-MS) is another powerful analytical technique that can be utilized for elemental mapping of both abiotic and biotic samples^37^; this second approach also has a lower detection limit (0.01 μg g^−1^) than XRF (0.1–1 μg g^−1^)^38^. In addition, X-ray absorption near-edge spectroscopy (XANES) is an element-specific method that can be used to analyze the *in vivo* ligand environments of metals within plants^39^. We utilized these analytical approaches to generate basic information on Cr accumulation and transportation mechanisms by exploring of Cr localization and speciation in *C. chinensis* Franch. Specifically, we used SR-μXRF mapping and LA-ICP-MS imaging to spatially locate Cr in tandem with XANES to the speciation of this metal. We also measured subcellular Cr concentrations using inductively coupled plasma mass spectrometry (ICP-MS) of different tissues to study the Cr subcellular distribution of *C. chinensis* Franch. Based on the above research, we could provide reliable reference data on the mechanisms of Cr accumulated by medicinal plants and on solving the high level of Cr in *C. chinensis* Franch. in the agricultural environment, so that further mitigates potential transfer to humans.

## Results

### Spatial imaging of Cr and other elements in roots and rhizomes using μ-XRF

**Figure 1:**
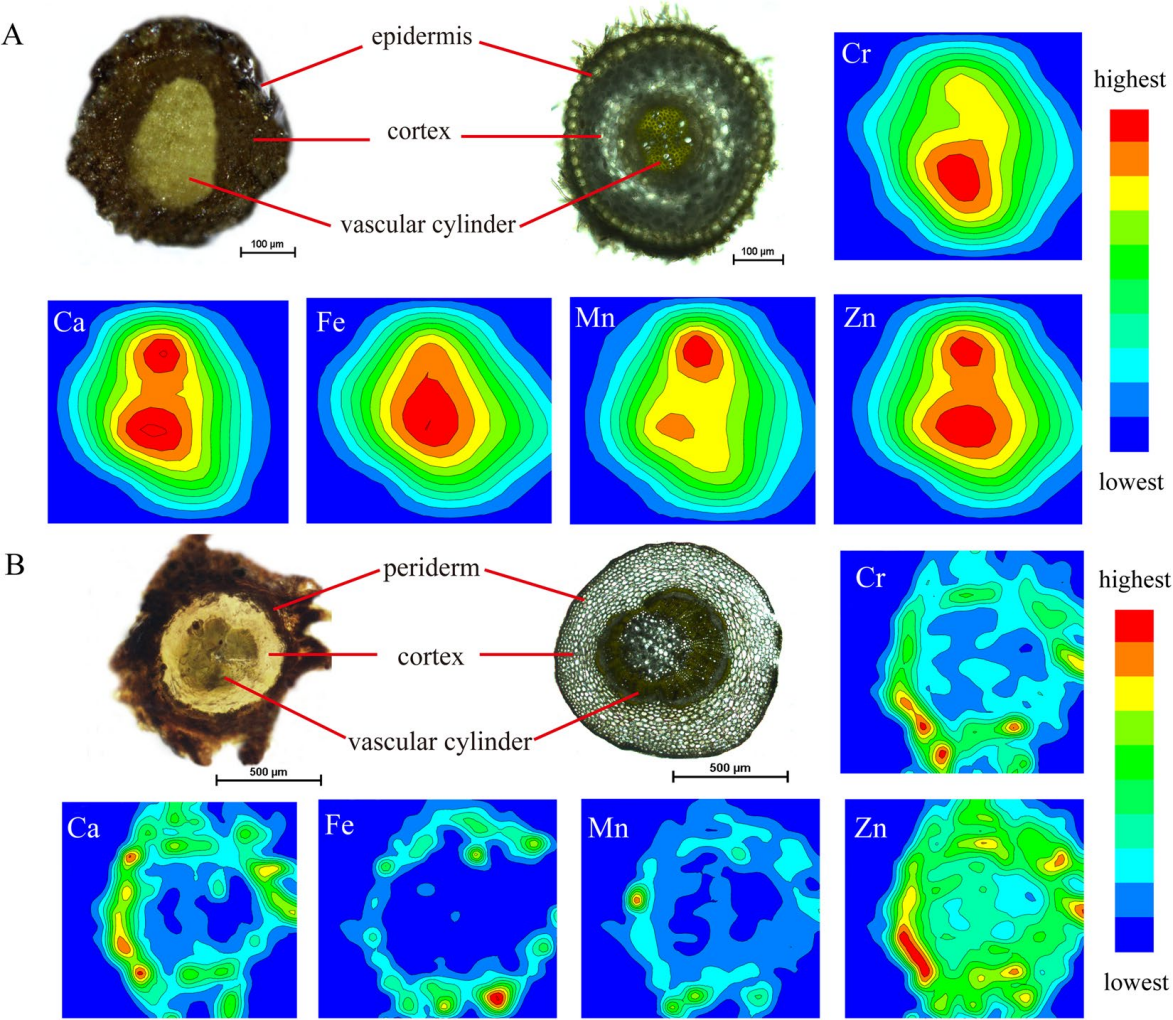
μ-XRF elemental maps for Cr, Ca, Fe, Mn, and Zn. Element distributions are shown in root (**A**) and rhizome (**B**) cross-sections of *C. chinensis* Franch. (the Cr10d group). The upper left images show the photographs of roots and rhizomes, the left image is the scanned sample cross-sections (300-μm thick) of roots and rhizomes, and the right image is the thin cross-sections (20-μm thick) for indicating the tissue structure. Each elemental map indicates the relative distribution of a specific element, but the count scales vary among maps. The color scale (red to blue) denotes normalized XRF intensities (highest to lowest).

In order to investigate the spatial distribution of Cr in *C. chinensis* Franch., we carried out XRF microscan mapping of plant roots and rhizomes. Thus, the integrated intensity of each element was calculated from the spectra and normalized to the intensities of the Compton scattering peaks. The elemental distribution maps of Cr, Fe, Mn, Ca, and Zn for scanned cross-sections of root and rhizome are shown in Fig. 1 alongside sample micrographs. Our μ-XRF maps of root cross-sections for the Cr10d group are presented in Fig. 1A; these results show that Cr is mainly localized in the central part of the root in these samples, a region that corresponds to the vascular cylinder. Similarly, maps show that the elements Fe, Mn, Ca, and Zn are all mainly localized within the central part of the root. μ-XRF mapping of Cr10d group rhizome cross-sections reveal that the Cr distribution pattern of this region differed (Fig. 1B); the main area of Cr accumulation in this case was the external layer (the periderm and some outer cortex) where Cr signals were of relatively higher intensity. Results showed that Ca, Fe, and Mn distribution patterns in rhizome cross-sections were similar to those of Cr, while Zn was distributed throughout the entire rhizome tissue in this part of the plant with higher levels recorded within the periderm.

### Spatial imaging of Cr and other elements in petioles using LA-ICP-MS

Because the leaves of *C. chinensis* Franch. have low Cr concentrations (Supplementary Table S2), we utilized LA-ICP-MS for spatial micromapping as this technique has a lower detection limit than XRF^38^. Thus, to obtain quantitative data, SRM NIST 1547 Peach Leaves was spiked with defined concentrations of various analytes and used for calibration and used SRM NIST 1570a (Trace Elements in Spinach Leaves) to validate the quantification procedure. The calibration curves yielded by these standards exhibited good linearity, with correlation coefficients ranging between 0.9961 (Mn) and 0.9991 (Ca) (Supplementary Fig. S1). Results show that the Cr concentration in peach leaves was 0.98 ± 0.03 μg g^−1^, compared to a certified concentration of 1 μg g^−1^. The concentrations of other elements of interest using LA-ICP-MS were also in agreement with certified values (Table 1).

**Table 1:** Comparison of analyte concentrations in NIST SRM 1547 Peach Leaves measured using LA-ICP-MS with the certified values.

	Cr	Ca	P	Mn	Cu
Certified value (μg g^−1^)	1	15,600 ± 200	1,370 ± 70	98 ± 3	3.7 ± 0.4
LA-ICP-MS (μg g^−1^)	0.98 ± 0.03	15,448 ± 22	1,355 ± 60	95.5 ± 0.4	3.7 ± 0.2

Mean ± standard deviation (replicates = 3).

**Figure 2:**
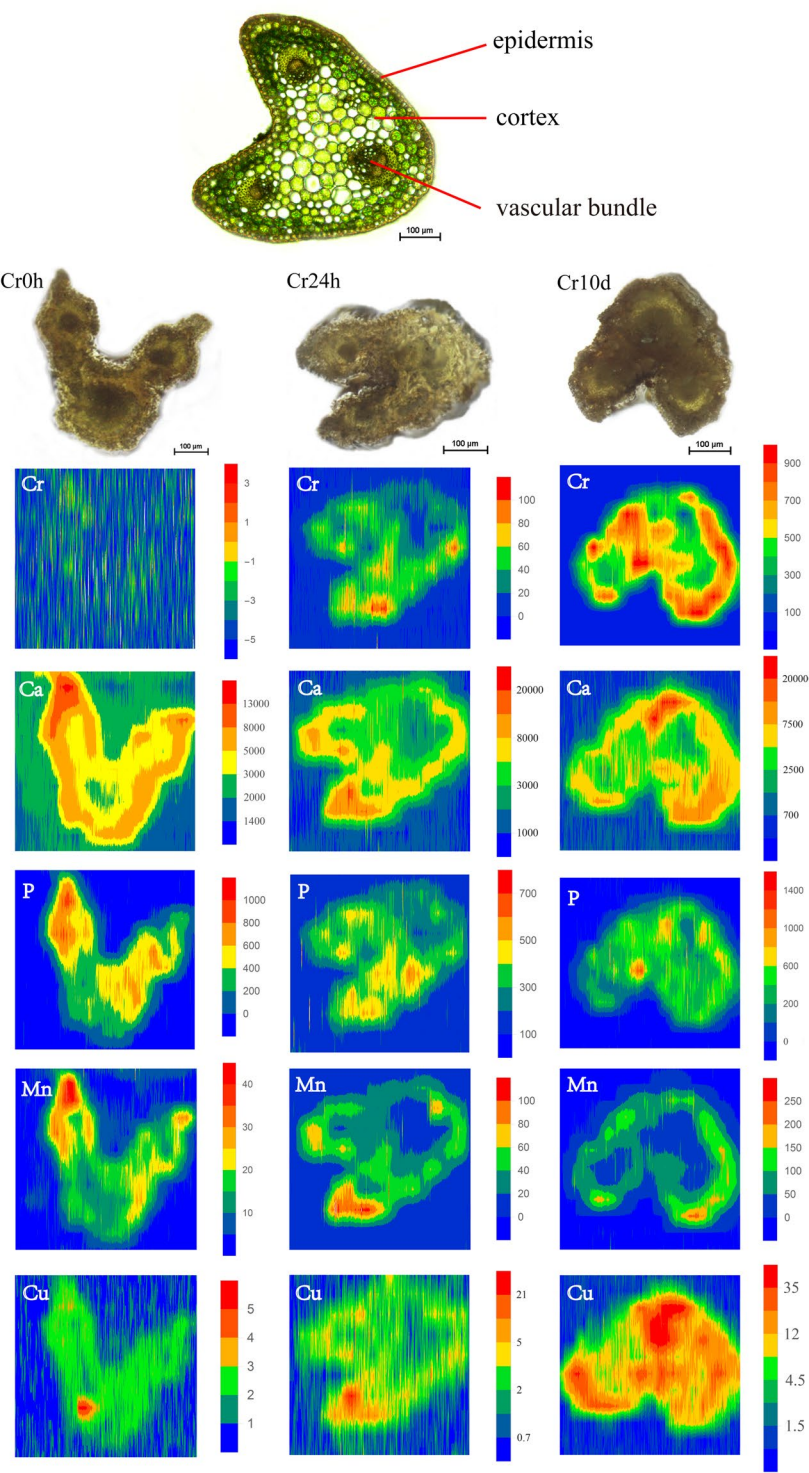
LA-ICP-MS elemental images of Cr, Ca, P, Mn, and Cu in petiole cross-sections of *C. chinensis* Franch. The upper image show the thin cross-sections (10-μm thick) for indicating the tissue structure of petioles, and the three images below it are the scanned sample cross-sections (300-μm thick) images of petioles from the Cr0h, Cr24h, and Cr10d group, respectively. Each elemental image indicates the relative distribution of a specific element. The color scale (red to blue; highest to lowest) denotes element concentrations.

We generated a series of two-dimensional elemental distributions profiles for *C. chinensis* Franch. petioles by scanning cross-sections line-by-line using a focused laser beam and analyzing them with mass spectrometry. After quantification, image processing was performed with the aid of the Wolfram Mathematica program 10 (Wolfram Research, USA). We quantitatively assessed the accumulation of Cr and other elements of interest (Ca, P, Mn, and Cu) using LA-ICP-MS imaging (Fig. 2).

Although our results reveal an uneven distribution of Cr across petiole cross-sections, images show that Cr is principally accumulated in the cortex as well as within some vascular bundles (Fig. 2). Images for Cr0h group petiole samples could not be created because recorded Cr concentrations were low in these regions. Images show that while Ca is also distributed across almost the whole cross-section of petioles, this element is preferentially accumulated in the cortex, as well as within some vascular bundles, similar to Cr. In addition, while the distribution of Mn is also quite similar to that of Ca. LA-ICP-MS images reveal that P and Cu are distributed throughout the whole petiole cross-sections.

Results show that the Cr concentrations in the petioles of the Cr24h group generally ranged 20 μg g^−1^–60 μg g^−1^, levels were greater than 100 μg g^−1^ in some places. Similarly, measured Cr levels within the Cr10d group generally ranged 400 μg g^−1^–700 μg g^−1^, rising to greater than 900 μg g^−1^ in some places. These results are consistent with subcellular distribution data; concentrations of Cr increased in concert with Cr treatment time in leaves (Supplementary Table S2). Compared to Cr0h petioles, Mn and Cu concentrations were higher in the Cr24h and Cr10d groups; data show that concentrations of Mn generally ranged 10 μg g^−1^–25 μg g^−1^, 20 μg g^−1^–60 μg g^−1^, and 50 μg g^−1^–150 μg g^−1^ in the Cr0h, Cr24h, and Cr10d groups, respectively, while concentrations of Cu in these groups were generally renged 2 μg g^−1^–3 μg g^−1^, 2 μg g^−1^–5 μg g^−1^, and 4.5 μg g^−1^–35 μg g^−1^, respectively.

### Cr speciation analysis of using XANES

**Figure 3:**
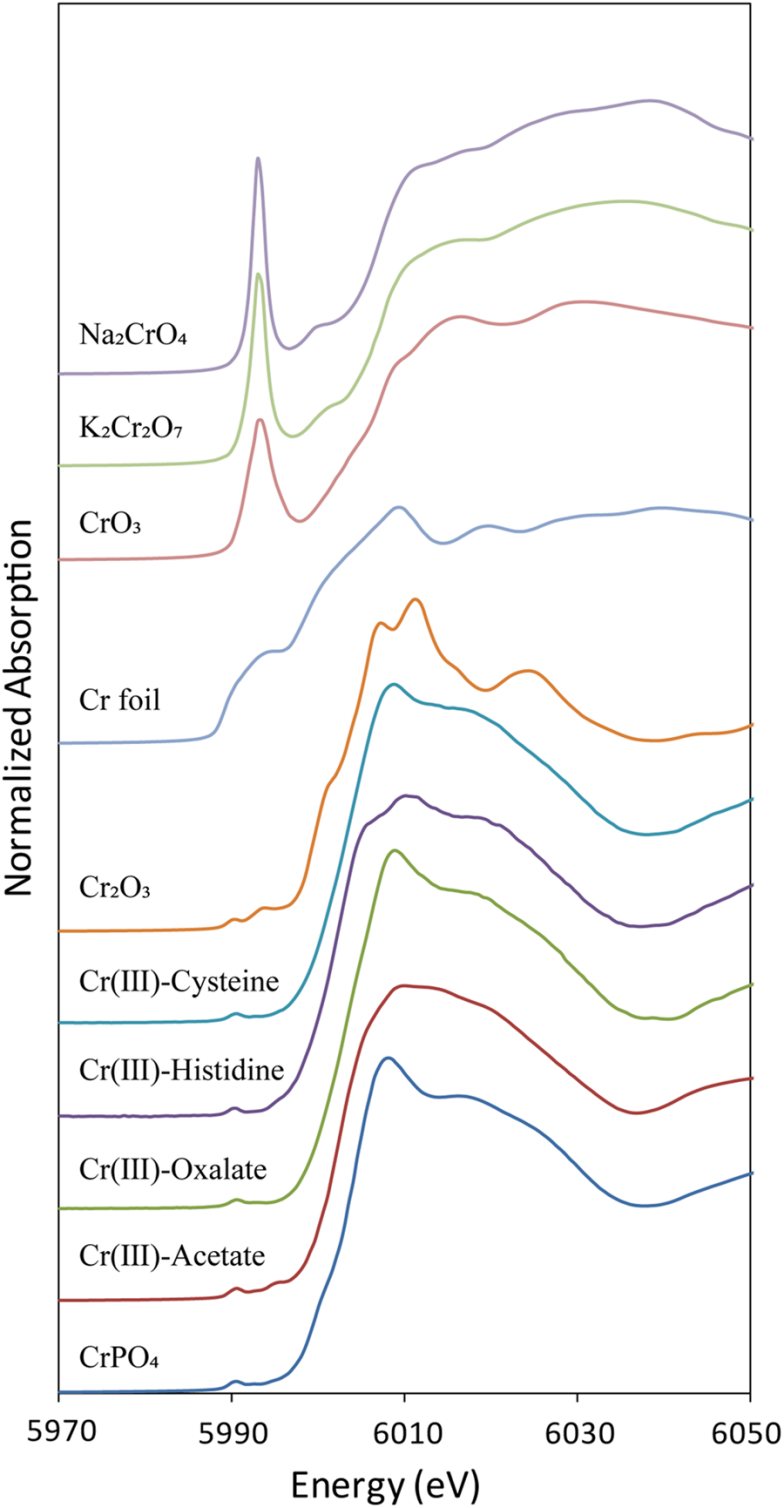
XANES data for the Cr(VI) reference compounds K_2_Cr_2_O_7_, Na_2_CrO_4_, and CrO_3_, the Cr(III) reference compounds Cr_2_O_3_, Cr(III)-oxalate, Cr(III)-acetate, Cr(III)-histidine, Cr(III)-cysteine, and CrPO_4_, and the Cr(0) reference compound Cr foil.

The XANES data generated in this study for the reference compounds are shown in Fig. 3. These results show that Cr(VI) compounds exhibit well-defined, large pre-edge peaks because of the 1s-to-3d electron transition; this is characteristic of the orbital mixing in four-coordinated Cr(VI). However, as Cr(III) forms complexes with six-fold octahedral coordination, the pre-edge peak in this case is markedly smaller and is located at a slightly lower energy^40^, while elemental chromium [Cr(0), Cr foil] exhibits a broad pre-edge conduction band^41^. Thus, comparison of the Cr Kα XANES spectra for Cr(VI), Cr(III), and Cr(0) compounds shows that XANES can potentially be used to determine the oxidation state of plant Cr, as mixtures of Cr(VI), Cr(III), and Cr(0) can generally be identified based on their unique pre-edge peak features.

**Figure 4:**
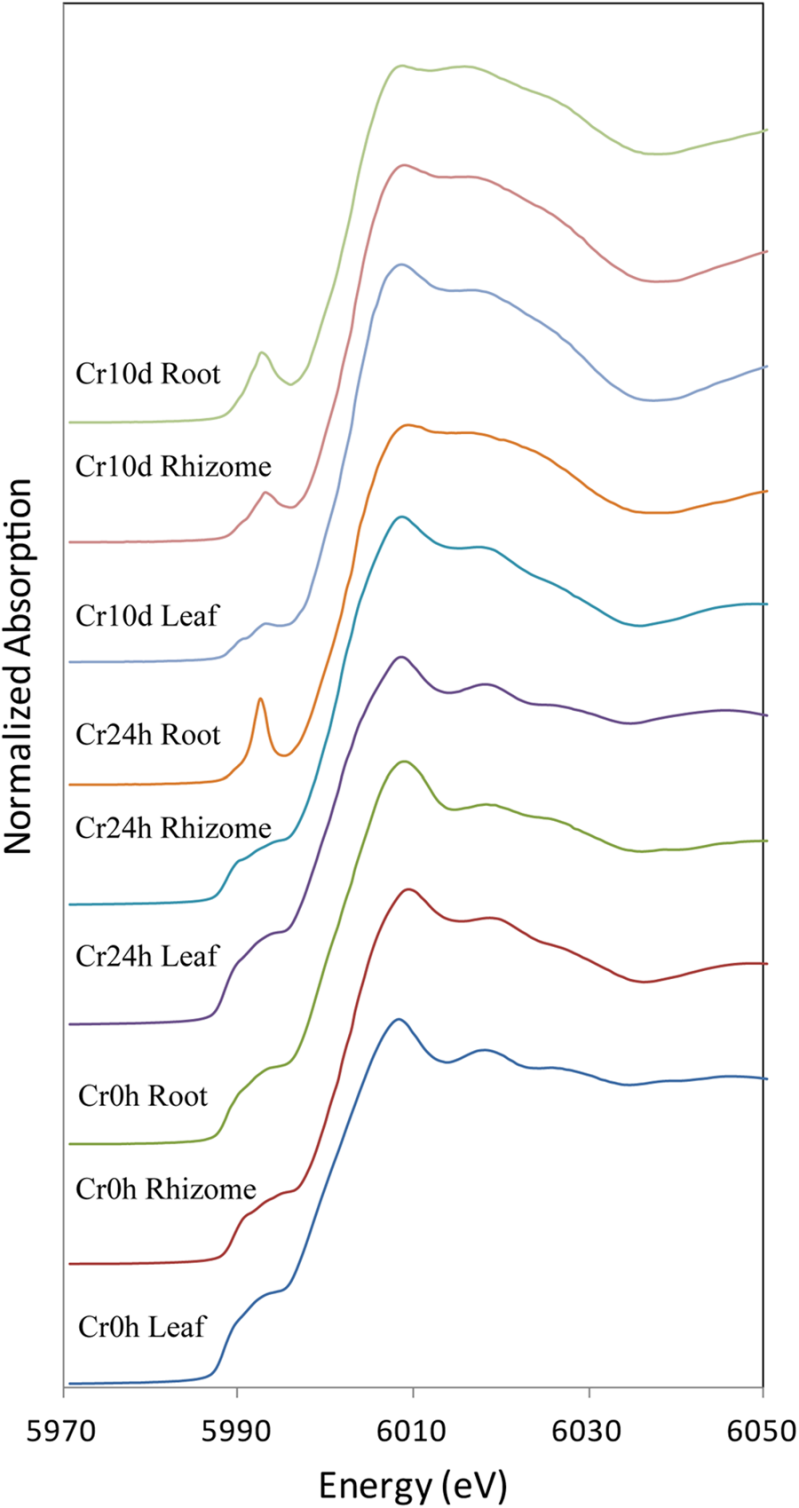
LCF-XANES data for *C. chinensis* Franch. roots, rhizomes, and leaves from the Cr0h, Cr24h, and Cr10d groups.

Figure 4 shows the sample spectra which has been compared to and fitted using the spectra of the reference compounds, while Cr K-edge XANES spectra of samples are presented in Supplementary Fig. S2. These results show that the pre-edge features of normalized Cr Kα spectra are superficially similar to those of the Cr foil in the case of plant samples from the Cr0h group, as well as rhizome and leaf samples from the Cr24h group. Root and rhizome spectra for the Cr10d group, as well as root spectra for the Cr24h group, have similar pre-edge features to those of Cr(VI) reference compounds. Results show that the pre-edge characteristics of Cr10d group leaf spectra are similar to those generated from Cr(VI), Cr(III), and Cr(0) reference compounds.

In order to further extract quantitative information regarding the speciation of Cr in *C. chinensis* Franch., we subjected the normalized XANES spectra generated in this study to linear combination fitting (LCF) using all possible combinations of Cr reference compounds (Table 2). The proportions of detailed Cr species in *C. chinensis* Franch. samples assessed using XANES LCF are presented in Supplementary Table S3. Results show that just the species Cr(III) and Cr(0) are present in the Cr0h group and that the latter comprises 50.5–72.9% of the total (Table 2). The species Cr(III) and Cr(0) are present in the leaves and rhizomes of the Cr24h group, while Cr(VI) also occurs in roots. In the Cr10d group, Cr(VI) and Cr(III) occur in roots and rhizomes, and Cr(0) is also found in leaves. These data show that Cr(III) proportions in each tissue within the Cr24h and Cr10d groups were higher than those in the Cr0h group, while the proportion of Cr(VI) in roots was higher than in rhizomes and leaves. Results reveal the presence of Cr(III)-histidine in most of the samples tested; compared with the Cr0h group, the proportions of this species increased significantly under conditions of Cr stress. Finally, Cr(III) was present only as CrPO_4_ and Cr(III)-histidine (mostly the former) in Cr24h group roots, as well as in various tissues of the Cr10d group.

**Table 2:** Proportions of Cr species in *C. chinensis* Franch. samples as assessed by Cr Kα XANES LCF analysis.

Sample		Cr species (%)			Sum	R-factor	Chi-square value
		Cr(0)	Cr(III)	Cr(VI)			
Cr0h	Leaf	72.9 ± 0.3	32.3 ± 0.4		105.2	0.000846	0.06851
	Rhizome	50.5 ± 0.6	53.1 ± 6.0		103.6	0.000212	0.01835
	Root	58.6 ± 1.2	46.2 ± 11.7		104.8	0.000475	0.04260
Cr24h	Leaf	69.7 ± 0.3	37.1 ± 0.4		106.8	0.001418	0.01913
	Rhizome	43.2 ± 0.4	60.1 ± 0.5		103.3	0.000310	0.02816
	Root		67.5 ± 4	31 ± 6.6	98.5	0.000143	0.01187
Cr10d	Leaf	10.1 ± 0.2	80.8 ± 0.9	10.7 ± 0.2	101.6	0.000119	0.01114
	Rhizome		75.3 ± 4.4	23.0 ± 1	98.3	0.000128	0.01117
	Root		63.6 ± 0.7	35.5 ± 0.3	99.1	0.000263	0.02190

### The subcellular distribution of Cr

**Figure 5:**
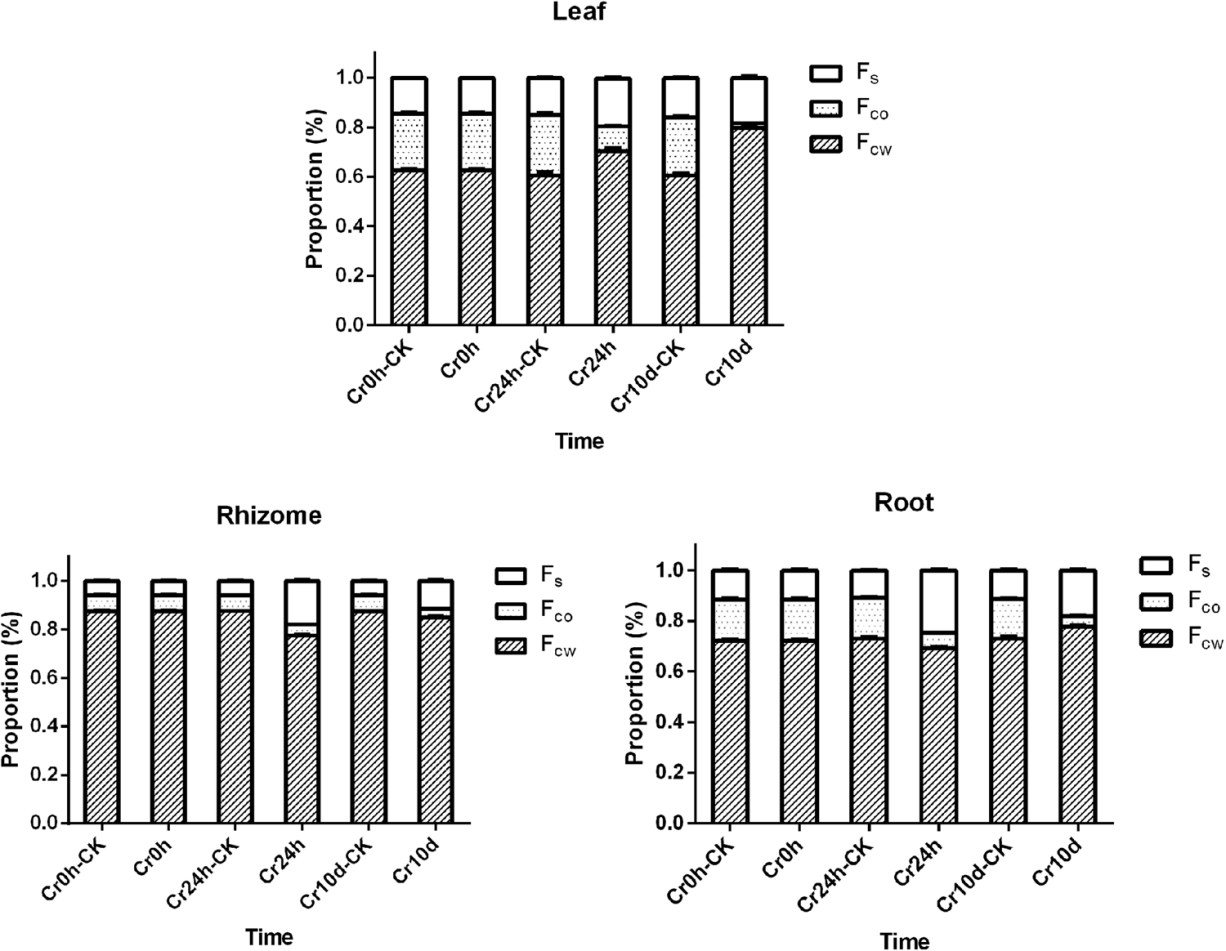
Effects of time of exposure to Cr(VI) on the subcellular distribution of Cr in the leaves, rhizomes, and roots. (replicates = 3). F_cw_, F_co_, and F_s_ respectively represent the proportion of Cr in cell walls, organelle-containing fractions, and the soluble fractions. The Cr proportion of each subcellular fraction is equal to the Cr content in each subcellular fraction divided by the total Cr content in tissues.

Our results show that the Cr concentrations in each *C. chinensis* Franch. tissues increased in concert with exposure time to Cr (Supplementary Table S2), and that concentrations were highest in roots, followed by rhizomes and leaves (Table 3). The effects of Cr(VI) exposure time on the subcellular distribution of Cr in the leaves, rhizomes, and roots are shown in Fig. 5 and Supplementary Table S4. These data show that approximately 60.4–87.5% of the total Cr is compartmentalized in the F_cw_ in all tissues. Similarly, Cr proportions of F_co_ (6.6–24.5%) in all control group tissues were higher than those of F_s_ (5.8–16.1%). However, the Cr proportions of F_co_ decreased in all Cr24h and Cr10d group tissues, and became the lowest of all, between 1.8% and 10.2%, while the proportions of F_s_ significantly increased to 11.5–24.8%.

**Table 3:** Concentrations of Cr (mg kg^−1^ fresh weight) in leaf, rhizome, and root of *C. chinensis* Franch. at different treatment time.

Tissues	Cr concentration (mg kg^−1^)		
	Cr0h	Cr24h	Cr10d
Leaf	0.379 ± 0.017^c^	0.729 ± 0.029^c^	10.587 ± 0.448^c^
Rhizome	1.151 ± 0.039^b^	3.060 ± 0.082^b^	34.129 ± 0.807^b^
Root	1.840 ± 0.047^a^	75.953 ± 2.354^a^	281.183 ± 8.369^a^

Mean ± standard deviation (replicates = 3). Values with different letters in the same column indicate a significant difference at *p* < 0.05.

## Discussion

The μ-XRF and LA-ICP-MS not only allow for the nondestructive spatial visualization of metal abundance, but also have the great advantage that multiple elements can be detected simultaneously^29,42–44^, while the latter has a lower detection limit (0.01 μg g^−1^) than XRF (0.1–1 μg g^−1^)^38^. Our imaging results show that Cr distribution patterns vary among tissues in *C. chinensis* Franch. In roots, Cr principally accumulates in the central vascular cylinder, while in rhizomes, Cr occurs mostly in external layers that correspond to the periderm and some outer cortex. Our LA-ICP-MS results show that Cr in petioles is mostly accumulated in the cortex and some vascular bundles. The Cr distribution patterns also varied in tissues of *G. pseudochina* (L.) DC.; in roots, relatively high-intensity Cr signals were detected in the periderm and the vascular bundle, while in stems, Cr was largely restricted to the vascular tissue, especially the xylem, when visualized using μ-XRF elemental maps of the cross-sections of the plant treated with Cr(VI)^34^. Previous studies have also shown that Cr mainly accumulated in root vascular cylinders; in *L. esculentum* Mill, for example, the Cr signal was higher in the root vascular cylinder than in either the cortex or surface^33^. Similarly, an X-ray analysis carried out on Mesquite revealed higher Cr deposition in root xylem and phloem structures^45^, while Cr was also detected in the root vascular bundles and cortex of *Taraxacum platypecidum*^46^.

Subsequent to absorption by roots, Cr is primarily transported through the xylem to the aerial parts of plants^21,47^ and must therefore cross the endodermis via symplast^11,21^. Our μ-XRF data suggest Cr enters the central vascular cylinder after absorption from the root; we therefore speculate that Cr may cross the endodermis via symplast within the root before being up-transported along the xylem and then principally accumulate in the rhizome periderm and some outer cortex, as well as in the cortex and some vascular bundles of petioles.

The μ-XRF mapping reveals correlations between Cr levels and those of other elements. For example, the Cr distribution in rhizomes is similar to that of Fe and Ca, and is also coincident with the Mn and Zn distribution in roots. Our LA-ICP-MS images also reveal a similar Cr distribution pattern to Ca and Mn; indeed, distributional similarities between Cr and Ca are seen in all tissues. A correlation between these two elements corroborates previous results for *G. pseudochina* (L.) DC^34^ and *Helianthus annuus* (sunflower)^48^ treated with Cr(VI).

As Cr is structurally similar to certain essential elements, it may affect plant mineral nutrition in a complex manner^11,19^. Liu *et al*. observed significant alterations in nutrient uptake when *Amaranthus viridis* L. was exposed to Cr stress^49^. Cr has been shown to influence both macro- and micronutrient uptake in paddies^50^. Our LA-ICP-MS results show that, compared with Cr0h group petioles, levels of both Cu and Mn markedly increased under Cr stress suggesting that Cr affected nutrient uptake by *C. chinensis* Franch. Concentrations of Cu have also been shown to increase in roots of *Matricaria chamomile*^51^ and significantly increase in stems of *Genipa americana*^52^ when these plants are subjected to Cr(VI) stress. However, Liu *et al*. also showed that Cr(VI) reduced Cu uptake in *A. viridis*^49^ and exposure to Cr(VI) led to a decline of Cu in *Citrullus vulgaris*^53^. Conflicting reports have also been published regarding the effects of Cr(VI) on Mn uptake; Cr(VI) has been shown to enhance Mn uptake in *C. vulgaris*^53^ and *Lolium perenne*^54^ but decrease Mn uptake in *Brassica oleracea*^55^ and *A. viridis*^49^. The causes and consequences of these complex effects will require further research.

Our results show that when grown with Cr(VI), Cr(VI) was absent in the leaves and rhizomes from the Cr24h group, and the proportions of Cr(III) in both Cr24h and Cr10d samples were all higher than those of the Cr0h group, increases of 1.15–2.50, 1.13–1.42, and 1.46–1.38 times in leaves, rhizomes and roots, respectively. These results indicated that a reduction of Cr(VI) to Cr(III) occurred when *C. chinensis* Franch. treated with Cr(VI). This phenomenon has also been observed in other plant species^18,29,34,40,56,57^, and is thought to represent an important detoxification pathway^18^. However, the mechanism by which Cr(VI) is reduced to Cr(III) and whether, or not, this takes places outside, or within the plant, remains unknown^56^. Once Cr(VI) traverses the plasma membrane, it has been suggested that plant-based Cr(VI) reduction is mediated by Fe(III)-reductase enzymes^19,52,58^; this is corroborated by the fact that the addition of Cr to Fe-deficient plants increases root-associated Fe(III)-reductase activity^59^ and enhances Fe availability^56,60^. In addition, chromate reductases have been isolated from bacteria^61^ and some researchers have hypothesized that plants may contain similar enzymes^32,61,62^. It has been suggested that while the reduction of Cr(VI) to Cr(III) mainly occurs inside the root cells, this can also take place in aerial parts of the plant^32^. The reduction of Cr(VI) to Cr(III) could also occur within the rhizosphere^31^.

The XANES LCF data demonstrate that Cr(VI) is present in the roots of Cr24h group plants as well as in all tissues in the Cr10d group; and the proportion of Cr(VI) in roots is higher than in either rhizomes or leaves. Plants can uptake both Cr(III) and Cr(VI)^7^. Howe *et al*. also reported on the presence of Cr(VI), Cr(V), and Cr(III) in subterranean clover plants treated with high concentrations of Cr(VI)^18^. The presence, or absence, of Cr(VI) and its translocation from roots to shoots may depend on the concentration of Cr(VI) in an experimental system^34^. Zayed *et al*. noted the presence of just Cr(III) in vegetable crops hydroponically treated with 1 mg L^−1^ of Cr(VI)^40^, while Cr(VI), Cr(V), and Cr(III) were all detected in the leaves of *G. pseudochina* (L.) DC. treated with a high concentration of Cr(VI) (100 mg L^−1^)^34^. These observations suggested that when the Cr(VI) level was low, plants may completely reduce Cr(VI) to Cr(III), while at higher concentrations, some Cr(VI) may remain within tissues^18^. We used a relatively high concentration of Cr(VI) (0.5 mM) in our experiments, and reported that the concentration of this element in tissues increased with exposure time and that the highest concentrations were in roots. These factors may explain why we still observed Cr(VI) within plants as well as the fact that highest proportion of Cr(VI) was recorded in roots.

We noted the presence of Cr(III)-histidine in most *C. chinensis* Franch. samples; the levels of this species increased significantly following Cr exposure (Supplementary Table S3), which suggests that histidine is a potential ligand in this plant. This observation is coincident with previously published X-ray absorption fine structure (XAFS) data for both alfalfa and mesquite that show that carboxyl groups play important roles in Cr(III) binding^57,63^. At the same time, amino acids like histidine serve as heavy metal ligands and play roles in metal tolerance and detoxification^64^; Krämer *et al*. found, for example, that free histidine functions as a Ni-transport ligand in the plant of genus Alyssum^65^, while Küpper *et al*. showed that histidine-complexed Zn is transported in stable form by the Zn-hyperaccumulator *Thlaspi caerulescens*^39^. In addition, Wu *et al*. reported that free histidine probably functions as the main Cu ligand in roots of *Elsholtzia splendens*^66^. The results of this study also show that Cr(III) is present mainly as CrPO_4_ (between 32.7% and 51.6%) in Cr24h group roots as well as in all Cr10d group tissues. This result corroborates the findings reported by Aldrich *et al*. who reported CrPO_4_ proportions of 65% and 55% in stems and leaves, respectively, following culture in agar containing Cr(VI)^57^. Previous XANES LCF data have also been used to demonstrate that CrPO_4_ is the predominant Cr(III) form in *Convolvulus arvensis* grown in Cr(VI)^67^. Additionally, Cr(III) was present only as CrPO_4_ and Cr(III)-histidine in Cr24h group roots, and in various tissues of the Cr10d group. Thus, both histidine and phosphate may play important roles in Cr accumulation and detoxification in *C. chinensis* Franch.

Our XANES LCF data reveal an interesting further result, that the medicinal plant *C. chinensis* Franch. contains the elemental chromium Cr(0) form (Table 2). This result is novel. Previous laboratory evidence has shown that plants^68–73^, fungi^74^, bacteria^75^, and algae^76^ can intra- and extra-cellularly transform readily reducible metals, including Au, Ag, Cu, Se, Hg, Pd, and Te, to their elemental states^72^. The synthesis of metal nanoparticles by plants has also been explored in previous work where alfalfa seedlings were found to synthesize Au and Ag nanoparticles after metal uptake from metal-enriched media^68,69^. Sharma *et al*.^71^ also used XAFS to show that the reduction of Au(III) ions to Au(0) inside *Sesbania drummondii* plant cells or tissues, alkaloids, and other secondary metabolites may serve as reducing agents and also to stabilize the nanoparticles within the cell^77^. Similarly, Manceau *et al*. used synchrotron microanalysis to show that both *Phragmites australis* and *Iris pseudoacorus* are assisted by endomycorrhizal fungi to transform Cu into metallic nanoparticles located in, or near to, roots when grown in contaminated soils^72^. Other researchers have also shown that Au nanoparticles form optimally at low pH values^70,78^. Thus, the presence of Cr(0) in *C. chinensis* Franch. may be attributable to the fact, first, that alkaloids are the principal secondary metabolites of this plant; second, that this species grows most optimally in acidic soils, and third, that associated microorganisms may directly, or indirectly, reduce Cr ions and stabilize the nanoparticles. Therefore, further research will be required to explore the mechanisms underlying the presence of Cr(0) in *C. chinensis* Franch. It may even be possible to develop nontoxic and environmentally friendly plant-based approaches for the synthesis of Cr nanoparticles.

Previous research has demonstrated that the subcellular distribution of Cr significantly affects the accumulation, migration, and detoxification of this element in a range of plant species and cultivars^79,80^. Figure 5 shows that Cr was primarily located in the cell walls of all the tissues tested in this study (60.4–87.5%) (Supplementary Table S4), indicating that Cr was largely retained in the cell walls. The plant cell wall is the first barrier for heavy metal movement into a cell^81^, and is composed principally of polyose (including cellulose, hemicelluloses, and pectins) and proteins that strongly bind metal ions^82^, thus restricting ion membrane transport to maintain normal cellular metabolism^83^. Numerous studies have indicated that the cell wall in many plants alleviates the toxicity of Cr and other heavy metals; Zeng *et al*. showed, for example, that the walls of rice root and leaf cells contained the largest proportions of Cr^84^.

Vacuoles are also major sites of heavy metal accumulation^85^; these are dynamic organelles that can comprise up to 90% of the total cellular volume^86^. We found that Cr proportions of F_s_ increased markedly in all Cr24h and Cr10d group tissues, up to 1.14–3.05 times of control group levels (Supplementary Table S4). This indicated that after crossing the plant cell walls, Cr ions entered into vacuoles to protect cellular activities when the plant was subjected to Cr stress. Vacuoles contain numerous metal ligands (i.e., proteins, organic acids, and bases), that chelate and compartmentalize metal ions to further protect the cell^87^; toxic metal cations that cross the plant cell walls are also sequestered predominantly within vacuoles^87^.

It is therefore clear that the cell wall was the principal Cr storage compartments in *C. chinensis* Franch., and after crossing the plant cell walls, Cr were sequestered predominantly within vacuoles when the plant was subjected to Cr stress, functioning to reduce the toxic effects of this metal. The results of this study strongly corroborate previous finding from Cr hyperaccumulator *Leersia hexandra*, 83.2% of the root Cr was localized in the cell wall fraction^88^. Researches have also shown that other heavy metals are dealt with in similar ways; Pb was accumulated predominantly in the cell walls of roots in the vegetable pakchoi^89^, while Cd was found principally in the cell wall of shoots in *Impatiens walleriana*^90^.

Our results show that Cr concentrations are higher in roots than in rhizomes and leaves (Table 3). This suggested that Cr preferentially accumulated in the roots of *C. chinensis* Franch., and corroborated earlier research that Cr concentrations in roots was higher than in the stems, leaves, and fruits of *L. esculentum* Mill^33^. This metal has also been shown to accumulate preferentially in chamomile roots with a translocation factor of less than 0.007^51^. Indeed, Cr is poorly translocated towards the aerial parts of most plant species, and is mainly retained in root tissues^91^; the concentration of Cr in roots is sometimes 100-times higher than in shoots^11^. Increased sequestration of Cr in plant roots is probably the result of the formation of insoluble Cr compounds within plants^7^. Some researchers have suggested that sequestration of Cr in root cells is because it is accumulated in vacuoles as a natural defense mechanism to limit its toxic potential^7,11,19,92^. It is possible that Cr was immobilized in root cell walls and vacuoles in *C. chinensis* Franch. after binding to proteins and organic acids rendering it less toxic. In addition, Srivastava *et al*. suggested that organic acids (carboxylic and amino acids) in root exudates might enhance Cr absorption by roots^93^.

The Cr localization and speciation information, obtained in this study using μ-XRF, LA-ICP-MS, ICP-MS, and XAFS provides additional evidence furthering our understanding of Cr transportation, accumulation, and tolerance in *C. chinensis* Franch. Our results demonstrated that Cr mainly accumulates in root vascular cylinders, in rhizome periderms and some outer cortex, and in petiole cortex and some vascular bundles. The preferential accumulation of Cr in cell walls as well as the reduction of Cr(VI) to Cr(III) alleviates Cr toxicity and may be a natural plant defense mechanism. It is also possible that both histidine and phosphate play important roles in Cr accumulation and detoxification in *C. chinensis* Franch. The new understanding of Cr accumulation presented in this study provides a theoretical basis for evaluating the Cr status in other medicinal plants, and for reducing the levels of Cr in *C. chinensis* Franch., so that further mitigates potential transfer to humans. The results of this study will also aid in the establishment of appropriate Cr-level standards for traditional Chinese medicines. Our new finding, that *C. chinensis* Franch. contains elemental chromium Cr(0), represents an small contribution to research on Cr in plants. Therefore, resolving the problem of high level of Cr in coptidis rhizoma, establishing reasonable Cr limit standard for traditional Chinese medicine, and exploring the mechanisms underlying the presence of Cr(0) in *C. chinensis* Franch. are important task for the future studies.

## Materials and Methods

### Plant cultures

We collected samples of *C. chinensis* Franch. from Zhenping County in the city of Ankang, Shaanxi Province, China. All samples were washed and were cultivated hydroponically in darkened and aerated containers in a solution containing 2 mM Ca^2+^, 7 mM NO^3+^, 3.5 mM K^+^, 0.5 mM H_2_PO_4_^−^, 1 mM Mg^2+^, 1 mM SO_4_^2−^, 10 μM H_3_BO_3_, 5 μM MnSO_4_, 0.5 μM ZnSO_4_, 0.1 μM CuSO_4_, 0.1 μM Na_2_MoO_4_ · 2H_2_O, 0.1 μM CoCl_2_ · 6H_2_O, and 20 μM Fe-EDTA. The pH was adjusted daily to pH 5.6 using 0.1 N NaOH or 0.1 N HCl. After 4 weeks of preculture, the plants were exposed to concentrations of Cr at 0 mM (CK) and 0.5 mM in the form of K_2_Cr_2_O_7_. Each treatment featured three replicates (15 plants each). The treatment concentration was based on the pre-experimental studies. Plants were harvested following exposure to Cr at 0 h (Cr0h), after 24 h (Cr24h) and 10 d (Cr10d), for studing the speciation changes of Cr in *C. chinensis* Franch. during the treatment. The detailed information of the pre-experiment was described in Supplementary information. Plants were grown under natural light, at day/night temperatures of 25 °C/20 °C, and under day/night humidities of 70%/85%, while the nutrient solution was renewed every 3 d.

### Elemental mapping of roots and rhizomes using μ-XRF

Roots and rhizomes were soaked in 20 mM Na_2_-EDTA for 15 min after harvesting to desorb surface-adsorbed Cr and were then washed with deionized water. We prepared sections (300-μm thick) using a cryotome (Leica; CM3050S, Germany) at −20 °C; these sections were placed on XRF tapes prior for μ-XRF analysis. Cr micromapping was carried out on beamline 4W1B at the Beijing Synchrotron Radiation Facility (BSRF), Beijing, China. The BSRF ran 2.5 GeV electron with current between 150 mA and 250 mA. The incident X-ray energy was monochromatized by W/B4C Double-Multilayer-Monochromator (DMM) operating at 15 keV and was focused down to a beam 50 μm in diameter by the polycapillary lens. The angle between the sample and the impinging beam was set at 45°. The Cr Kα (5,989 eV) fluorescence counts for all XRF microprobe experiments were collected at each step of a two-dimensional matrix (x step, 70–100 μm; y step, 50–100 μm). The step size used in each case depended on the area of each investigated sample. An Si (Li) solid state detector was used to detect X-ray fluorescence emission lines with live time of 15–30 s. The intensity of X-ray emission was proportional to the element content. The Kα fluorescence counts of elements Ca, Mn, Fe, Zn, and Cr were collected simultaneously. Three independent samples were mapped. Data reduction and processing were performed with the aid of the PyMCA package^94^. Elemental maps of tissue sections were generated using Origin 8.0 software (Originlab Corp., USA).

### Elemental imaging of petioles using LA-ICP-MS

Petiole cross-sections and standard reference materials (SRM) [National Institute of Standards and Technology (NIST) 1547 Peach Leaves and NIST 1570a Trace Elements in Spinach Leaves] were subjected to laser ablation (Geolas LA, Coherent, USA) coupled with a Quadrupole ICP-MS (Agilent 7700a ICP-MS, USA) to enable *in situ* microquantitative analysis. The details of our sample preparation as well as the standard compounds we used are described in Supplementary Material. The 193-nm UV laser beam emitted by the LA Compex102 laser unit (Lambda Physik, Germany) was subjected to homogenization to generate a flat-topped laser beam. Thus, a laser-ablated spot with a diameter of 44 μm was continuously walked across samples at a speed of 10 μm s^−1^. Our data collection was therefore continuous as between eight and nine data were generated for each designated element per second. There was an in-house homogenizer between the sample cell and ICP torch used to obtain a smooth signal. Laser-ablated materials were transported using high-purity helium gas (99.9995%) to the inductively coupled plasma, and the time-resolved analysis (TRA) mode was enabled for all measurements. We processed the SRM samples first before our tissue sections were scanned line-by-line using a focused laser beam; this procedure means that each sample is associated with a unique calibration curve to ensure data accuracy. As the initial laser coupling signal created instability, the first 20 s of each ablation signal was ignored upon analytical signal integration. The ICP-MS instrument settings were optimized by minimizing the oxide and low double-charge ratios while maximizing the stable signal intensities of all SRM and experimental samples. Other instrumental parameters are summarized in Table 4.

**Table 4:** The LA-ICP-MS operating conditions and data acquisition parameters.

**Geolas laser ablation system**			
Laser wavelength (nm)	193	Laser frequency (Hz)	6
Energy density (J cm^−2^)	13	Spot diameter (μm)	44
Ablation mode	Scanning-line-by-line	Distance between lines (μm)	50
Scan speed (μm s^−1^)	10		
**ICP-MS**			
RF power (W)	1,340	He carrier gas (L min^−1^)	0.8
Carrier gas (L min^−1^)	1.0	Omega lens (V)	12.2
Omega bias (V)	−110	Extraction 1 (V)	0
Extraction 2 (V)	−200	Plate bias (V)	−60
Scanning mode	Peak-hopping	Deflection (V)	14.2
Sample depth (mm)	6.1		
Signal monitored	^55^Mn^+^, ^63^Cu^+^, ^31^P^+^, ^43^Ca^+^, ^53^Cr^+^		

### XANES analysis

The freeze-dried samples and Cr reference compounds, contained Cr(VI) in the form of K_2_Cr_2_O_7_, Na_2_CrO_4_, and CrO_3_; Cr(III) in the form of Cr(III)-oxalate, Cr(III)-acetate, Cr(III)-histidine, Cr(III)-cysteine, CrPO_4_, and Cr_2_O_3_; and Cr(0) (elemental chromium) as a Cr foil, all were taken on beamline 1W1B at the BSRF for XANES analysis of the Cr K edge (5,989 eV). The ring storage energy of the synchrotron radiation accelerator during spectrum collection was 2.5 GeV while the current intensity was 150–250 mA. We used a Si (111) double-crystal monochromator (220, at *φ*90) with 1-mm wide entrance slit for all measurements. All XANES spectra for the Cr reference compounds were acquired in the transmission mode, while those for the experimental samples were collected in the fluorescence mode using a 19-element Ge solid-state detector (SSD) with a Cr foil to calibrate X-ray energy. Detailed information on preparation of sample and reference compounds is presented in the Supplementary Material to this paper. The XANES regions were then extracted from 5,970–6,050 eV of the entire spectra, processed and analyzed using ATHENA software (version 2.1.1). The minimal R-factor, chi-square, and reduced chi-square test were used to control the quality of data fitting.

### Subcellular distribution of Cr

We homogenized frozen fresh roots, rhizomes, and leaves were homogenized in a pre-chilled extraction buffer containing 50 mM Tris-HCl (pH 7.5), 250 mM sucrose and 1.0 mM C_4_H_10_O_2_S_2_ (DTE, Aladdin, USA). Cells were then separated into three fractions: cell walls, organelles, and soluble fraction (including vacuoles) using the differential centrifugation technique outlined by Wsigel and Jäger^95^ with some modifications. Homogenates were then transferred to centrifuge tubes and centrifuged at 3,000 rpm for 15 min. The precipitate was then designated the cell wall fraction (F_cw_) which mainly comprised cell walls and associated debris. The supernatant was then further centrifuged at 12,000 rpm for 45 min, the resultant pellet and supernatant comprised the organelle-containing fraction (F_co_) and the soluble fraction (F_s_), respectively. All operations were performed at 4 °C. We measured the Cr concentrations in the three fractions using ICP-MS. The data obtained in this experiment were subjected to a one-way analysis of variance (ANOVA), and an LSD test was performed to determine the significance of the difference between the mean values using SPSS software (SPSS Inc., Chicago, IL, Version 23.0). A value of P < 0.05 was considered statistically significant. Figures were performed using GraphPad Prism 6 software (GraphPad Software, San Diego, USA).

### Data availability statement

Additional supporting data to the article can be found in the supplementary material. The datasets generated during and/or analysed during the current study are available from the corresponding author on reasonable request.

## Electronic supplementary material

Supplementary Information

## References

[1] Tang J (2009). Berberine and Coptidis rhizoma as novel antineoplastic agents: a review of traditional use and biomedical investigations. Journal of ethnopharmacology.

[2] Liu Q (2011). Selective separation of structure-related alkaloids in Rhizoma coptidis with “click” binaphthyl stationary phase and their structural elucidation with liquid chromatography-mass spectrometry. The Analyst.

[3] Wang L (2014). New enantiomeric isoquinoline alkaloids from Coptis chinensis. Phytochemistry Letters.

[4] Zhao M, Xian YF, Ip SP, Fong HH, Che CT (2010). A new and weakly antispasmodic protoberberine alkaloid from Rhizoma Coptidis. Phytotherapy research: PTR.

[5] Chin LW (2010). Anti-herpes simplex virus effects of berberine from Coptidis rhizoma, a major component of a Chinese herbal medicine, Ching-Wei-San. Archives of virology.

[6] Wang J, Chen C (2009). Biosorbents for heavy metals removal and their future. Biotechnology Advances.

[7] Shahid M (2017). Chromium speciation, bioavailability, uptake, toxicity and detoxification in soil-plant system: A review. Chemosphere.

[8] Mombo S (2016). Management of human health risk in the context of kitchen gardens polluted by lead and cadmium near a lead recycling company. Journal of Soils & Sediments.

[9] Xiong TT (2016). Measurement of metal bioaccessibility in vegetables to improve human exposure assessments: field study of soil–plant–atmosphere transfers in urban areas, South China. Environmental Geochemistry & Health.

[10] Noli F, Tsamos P (2016). Concentration of heavy metals and trace elements in soils, waters and vegetables and assessment of health risk in the vicinity of a lignite-fired power plant. Science of the Total Environment.

[11] Shanker AK, Cervantes C, Loza-Tavera H, Avudainayagam S (2005). Chromium toxicity in plants. Environment international.

[12] Ashraf, A. *et al*. Chromium (VI) sorption efficiency of acid-activated banana peel over organo-montmorillonite in aqueous solutions. *International Journal of Phytoremediation* (2017). 10.1080/15226514.2016.1256372 27849143

[13] Butera S, Trapp S, Astrup TF, Christensen TH (2015). Soil retention of hexavalent chromium released from construction and demolition waste in a road-base-application scenario. Journal of hazardous materials.

[14] Chen T, Chang Q, Liu J, Clevers JG, Kooistra L (2016). Identification of soil heavy metal sources and improvement in spatial mapping based on soil spectral information: A case study in northwest China. Science of the Total Environment.

[15] Lilli MA, Moraetis D, Nikolaidis NP, Karatzas GP, Kalogerakis N (2015). Characterization and mobility of geogenic chromium in soils and river bed sediments of Asopos basin. Journal of hazardous materials.

[16] Bai, J. *et al*. Chromium exposure and incidence of metabolic syndrome among American young adults over a 23-year follow-up: the CARDIA Trace Element Study. *Scientific Reports* **5** (2015). 10.1038/srep15606 PMC4614983 26489690

[17] Reale L (2016). Cyto-histological and morpho-physiological responses of common duckweed (Lemna minor L.) to chromium. Chemosphere.

[18] Howe JA, Loeppert RH, Derose VJ, Hunter DB, Bertsch PM (2003). Localization and speciation of chromium in subterranean clover using XRF, XANES, and EPR spectroscopy. Environmental Science & Technology.

[19] Singh HP, Mahajan P, Kaur S, Batish DR, Kohli RK (2013). Chromium toxicity and tolerance in plants. Environmental Chemistry Letters.

[20] Panda SK (2003). Heavy metal phytotoxicity induces oxidative stress in Taxithelium sp. Current Science.

[21] Hayat S (2011). Physiological changes induced by chromium stress in plants: an overview. Protoplasma.

[22] de Oliveira LM (2016). Sulfate and chromate increased each other’s uptake and translocation in As-hyperaccumulator Pteris vittata. Chemosphere.

[23] de Oliveira LM, Ma LQ, Santos JA, Guilherme LR, Lessl JT (2014). Effects of arsenate, chromate, and sulfate on arsenic and chromium uptake and translocation by arsenic hyperaccumulator Pteris vittata L. Environmental pollution.

[24] Gardea-Torresdey JL, Peralta-Videa JR, Montes M, Rosa GDL, Corral-Diaz B (2004). Bioaccumulation of cadmium, chromium and copper by Convolvulus arvensis L.: impact on plant growth and uptake of nutritional elements. Bioresour Technol.

[25] Zhang, H. Behaviors of Trace Metals in Environment: *The Pollution in Regional and Metropolis Areas* (2014).

[26] Fang Q, Zhang H, Li K (2002). Contents of some trace elements in soil and medicinal materials of Coptis chinensis Franch. West China Journal of Pharmaceutical Sciences.

[27] Wang Y, He S, Wu Y (2007). Study on trace elements in coptis chinensis from Mount Emei. Lishizhen Medicine and Materia Medica Research.

[28] Arruda MAZ, Azevedo RA (2009). Metallomics and chemical speciation: towards a better understanding of metal-induced stress in plants. Annals of Applied Biology.

[29] Punshon T, Guerinot ML, Lanzirotti A (2009). Using synchrotron X-ray fluorescence microprobes in the study of metal homeostasis in plants. Annals of Botany.

[30] Hall JL (2002). Cellular mechanisms for heavy metal detoxification and tolerance. Journal of experimental botany.

[31] Bluskov S, Arocena JM, Omotoso OO, Young JP (2005). Uptake, distribution, and speciation of chromium in Brassica juncea. International Journal of Phytoremediation.

[32] Cervantes C, Devars S, Loza-Tavera H, Moreno-Sánchez R (2001). Interactions of chromium with microorganisms and plants. Fems Microbiology Reviews.

[33] Mangabeira P (2006). Chromium localization in plant tissues of Lycopersicum esculentum Mill using ICP-MS and ion microscopy (SIMS). Applied Surface Science.

[34] Mongkhonsin B, Nakbanpote W, Nakai I, Hokura A, Jearanaikoon N (2011). Distribution and speciation of chromium accumulated in Gynura pseudochina (L.) DC. Environmental and Experimental Botany.

[35] Augustynowicz J (2014). Chromium distribution in shoots of macrophyte Callitriche cophocarpa Sendtn. Planta.

[36] Chen YL (2014). Biosorption of Cr (VI) by Typha angustifolia: mechanism and responses to heavy metal stress. Bioresour Technol.

[37] Hu Z (2008). A local aerosol extraction strategy for the determination of the aerosol composition in laser ablation inductively coupled plasma mass spectrometry. Journal of Analytical Atomic Spectrometry.

[38] Mcrae R, Bagchi P, Sumalekshmy S, Fahrni CJ (2009). *In Situ* Imaging of Metals in Cells and Tissues. Chemical Reviews.

[39] Kupper H (2004). Tissue- and Age-Dependent Differences in the Complexation of Cadmium and Zinc in the Cadmium/Zinc Hyperaccumulator Thlaspi caerulescens (Ganges Ecotype) Revealed by X-Ray Absorption Spectroscopy. Plant physiology.

[40] Zayed A, Lytle CM, Qian JH, Terry N (1998). Chromium accumulation, translocation and chemical speciation in vegetable crops. Planta.

[41] Arcon I, Mirtic B, Kodre A (2005). Determination of Valence States of Chromium in Calcium Chromates by Using X-ray Absorption Near-Edge Structure (XANES) Spectroscopy. Journal of the American Ceramic Society.

[42] Hokura A (2006). Arsenic distribution and speciation in an arsenic hyperaccumulator fern by X-ray spectrometry utilizing a synchrotron radiation source. Journal of Analytical Atomic Spectrometry.

[43] Tian S (2010). Spatial imaging and speciation of lead in the accumulator plant Sedum alfredii by microscopically focused synchrotron X-ray investigation. Environmental Science & Technology.

[44] Tian SK (2009). Stem and leaf sequestration of zinc at the cellular level in the hyperaccumulator Sedum alfredii. The New phytologist.

[45] Arias JA (2010). Effects of Glomus deserticola inoculation on Prosopis: Enhancing chromium and lead uptake and translocation as confirmed by X-ray mapping, ICP-OES and TEM techniques. Environmental and Experimental Botany.

[46] Wu S (2016). Chromium immobilization by extraradical mycelium of arbuscular mycorrhiza contributes to plant chromium tolerance. Environmental and Experimental Botany.

[47] Shahid M (2017). Foliar heavy metal uptake, toxicity and detoxification in plants: A comparison of foliar and root metal uptake. Journal of hazardous materials.

[48] Kötschau A (2013). Mapping of macro and micro elements in the leaves of sunflower (Helianthus annuus) by Laser Ablation–ICP–MS. Microchemical Journal.

[49] Liu D, Zou J, Wang M, Jiang W (2008). Hexavalent chromium uptake and its effects on mineral uptake, antioxidant defence system and photosynthesis in Amaranthus viridis L. Bioresource Technology.

[50] Sundaramoorthy P, Chidambaram A, Ganesh KS, Unnikannan P, Baskaran L (2010). Chromium stress in paddy: (i) nutrient status of paddy under chromium stress; (ii) phytoremediation of chromium by aquatic and terrestrial weeds. Comptes rendus biologies.

[51] Kováčik J, Babula P, Hedbavny J, Klejdus B (2014). Hexavalent chromium damages chamomile plants by alteration of antioxidants and its uptake is prevented by calcium. Journal of hazardous materials.

[52] Santana KB (2012). Physiological analyses of Genipa americana L. reveals a tree with ability as phytostabilizer and rhizofilterer of chromium ions for phytoremediation of polluted watersheds. Environmental & Experimental Botany.

[53] Dube BK, Tewari K, Chatterjee J, Chatterjee C (2003). Excess chromium alters uptake and translocation of certain nutrients in citrullus. Chemosphere.

[54] Vernay P, Gauthiermoussard C, Hitmi A (2007). Interaction of bioaccumulation of heavy metal chromium with water relation, mineral nutrition and photosynthesis in developed leaves of Lolium perenne L. Chemosphere.

[55] Chatterjee J, Chatterjee C (2000). Phytotoxicity of cobalt, chromium and copper in cauliflower. Environmental pollution.

[56] Pradas del Real AE, Perez-Sanz A, Lobo MC, McNear DH (2014). The chromium detoxification pathway in the multimetal accumulator Silene vulgaris. Environ Sci Technol.

[57] Aldrich MV, Gardea-Torresdey JL, Peralta-Videa JR, Parsons JG (2003). Uptake and reduction of Cr(VI) to Cr(III) by mesquite (Prosopis spp.): chromate-plant interaction in hydroponics and solid media studied using XAS. Environmental Science & Technology.

[58] Zayed AM, Terry N (2003). Chromium in the environment: factors affecting biological remediation. Plant and Soil.

[59] Schmidt W (1996). Influence of chromium(lll) on root-associated Fe(lll) reductase in Plantago lanceolata L. Journal of experimental botany.

[60] Bonet A, Poschenrieder C, Barcelo J (1991). Chromium III-iron interaction in Fe-deficient and Fe-sufficient bean plants. I. Growth and nutrient content. Journal of Plant Nutrition.

[61] Opperman DJ, Van HE (2008). A membrane-associated protein with Cr(VI)-reducing activity from Thermus scotoductus SA-01. Fems Microbiology Letters.

[62] Mel Lytle C (1998). Reduction of Cr(VI) to Cr(III) by Wetland Plants:Potential for *In Situ* Heavy Metal Detoxification. Environmental Science & Technology.

[63] Tiemann KJ (1999). Use of X-ray Absorption Spectroscopy and Esterification to Investigate Cr(III) and Ni(II) Ligands in Alfalfa Biomass. Environmental Science & Technology.

[64] Salt DE, Prince RC, Baker AJM, Raskin I, Pickering IJ (2016). Zinc ligands in the metal hyperaccumulator Thlaspi caerulescens as determined using X-ray absorption spectroscopy. Environmental Science & Technology.

[65] Krämer U, CotterHowells JD, Charnock JM, Baker AJM, Smith JAC (1996). Free histidine as a metal chelator in plants that accumulate nickel. Nature.

[66] Wu B, Susnea I, Chen Y, Przybylski M, Becker JS (2011). Study of metal-containing proteins in the roots of Elsholtzia splendens using LA-ICP-MS and LC–tandem mass spectrometry. International Journal of Mass Spectrometry.

[67] Montes-Holguin MO (2006). Biochemical and spectroscopic studies of the response of Convolvulus arvensis L. to chromium(III) and chromium(VI) stress. Environmental Toxicology & Chemistry.

[68] GardeaTorresdey JL (2003). Formation and Growth of Au Nanoparticles inside Live Alfalfa Plants. Nano Letters.

[69] Gardea-Torresdey JL (2016). Alfalfa Sprouts:A Natural Source for the Synthesis of Silver Nanoparticles. Langmuir.

[70] Armendariz V (2004). Size controlled gold nanoparticle formation by Avena sativa biomass: use of plants in nanobiotechnology. Journal of Nanoparticle Research.

[71] Sharma NC (2007). Synthesis of plant-mediated gold nanoparticles and catalytic role of biomatrix-embedded nanomaterials. Environmental Science & Technology.

[72] Manceau A (2016). Formation of Metallic Copper Nanoparticles at the Soil−Root Interface. Environmental Science & Technology.

[73] Marshall AT (2009). Accumulation of gold nanoparticles in Brassic juncea. International Journal of Phytoremediation.

[74] Gadd GM (2007). Geomycology: biogeochemical transformations of rocks, minerals, metals and radionuclides by fungi, bioweathering and bioremediation. Mycological Research.

[75] Ha C (2015). Biorecovery of palladium as nanoparticles by Enterococcus faecalis and its catalysis for chromate reduction. Chemical Engineering Journal.

[76] Greene B (1986). Interaction of gold(I) and gold(III) complexes with algal biomass. Environmental Science & Technology.

[77] Powell RG, Smith CR (1981). An investigation of the antitumor activity of Sesbania drummondii. Journal of Natural Products.

[78] Gardea-Torresdey JL, Tiemann KJ, Parsons JG, Gamez G, Yacaman MJ (2002). Characterization of trace level Au(III) binding to alfalfa biomass (Medicago sativa) by GFAAS. Advances in Environmental Research.

[79] Qiu Q, Wang Y, Yang Z, Yuan J (2011). Effects of phosphorus supplied in soil on subcellular distribution and chemical forms of cadmium in two Chinese flowering cabbage (Brassica parachinensis L.) cultivars differing in cadmium accumulation. Food and chemical toxicology: an international journal published for the British Industrial Biological Research Association.

[80] Xin J, Huang B, Yang Z, Yuan J, Zhang Y (2013). Comparison of cadmium subcellular distribution in different organs of two water spinach (Ipomoea aquatica Forsk.) cultivars. Plant and Soil.

[81] Allan DL, Jarrell WM (1989). Proton and copper adsorption to maize and soybean root cell walls. Plant Physiology.

[82] Haynes RJ (1980). Ion exchange properties of roots and ionic interactions within the root apoplasm: their role in ion accumulation by plants. The Botanical Review.

[83] Peng HY, Yang XE, Tian SK (2005). Accumulation and ultrastructural distribution of copper in Elsholtzia splendens. Journal of Zhejiang University. Science. B.

[84] Zeng F (2011). Subcellular distribution and chemical forms of chromium in rice plants suffering from different levels of chromium toxicity. Journal of Plant Nutrition and Soil Science.

[85] Liu X (2015). Effect of applied sulphur on the uptake by wheat of selenium applied as selenite. Plant and Soil.

[86] Pittman JK (2005). Managing the manganese: molecular mechanisms of manganese transport and homeostasis. The New phytologist.

[87] Krämer U, Pickering IJ, Prince RC, Raskin I, Salt DE (2000). Subcellular localization and speciation of nickel in hyperaccumulator and non-accumulator Thlaspi species. Plant physiology.

[88] Liu J, Duan C-Q, Zhang X-H, Zhu Y-N, Hu C (2009). Subcellular distribution of chromium in accumulating plant Leersia hexandra Swartz. Plant and Soil.

[89] Wu Z, McGrouther K, Chen D, Wu W, Wang H (2013). Subcellular distribution of metals within Brassica chinensis L. in response to elevated lead and Chromium Stress. Journal of agricultural and food chemistry.

[90] Lai HY (2015). Subcellular distribution and chemical forms of cadmium in Impatiens walleriana in relation to its phytoextraction potential. Chemosphere.

[91] Jaison S, Muthukumar T (2016). Chromium Accumulation in Medicinal Plants Growing Naturally on Tannery Contaminated and Non-contaminated Soils. Biological Trace Element Research.

[92] Duarte B, Silva V, Caçador I (2012). Hexavalent chromium reduction, uptake and oxidative biomarkers in Halimione portulacoides. Ecotoxicology and Environmental Safety.

[93] Srivastava S, Prakash S, Srivastava MM (1999). Chromium mobilization and plant availability – the impact of organic complexing ligands. Plant and Soil.

[94] Sol (2007). A multiplatform code for the analysis of energy-dispersive X-ray fluorescence spectra. Spectrochimica Acta Part B Atomic Spectroscopy.

[95] Weigel HJ, Jager HJ (1980). Subcellular distribution and chemical form of cadmium in bean plants. Plant physiology.

