# Supplementary material for: Localization and Speciation of Chromium in Coptis chinensis Franch. using Synchrotron Radiation X-ray Technology and Laser Ablation ICP-MS
Source: Sci Rep. 2018 Jun 5;8:8603. doi: 10.1038/s41598-018-26774-x (PMC5988735; doi:10.1038/s41598-018-26774-x)
Supplement: Supplementary file 1 — Supplementary Information [file 41598_2018_26774_MOESM1_ESM.pdf]

Supplementary Information

Localization and Speciation of Chromium in *Coptis chinensis* Franch. using Synchrotron Radiation X-ray Technology and Laser Ablation ICP-MS

Wenli Huang[1], Jie Jiao[1], Mei Ru[2], Zhenqing Bai[1], Honglin Yuan[3], Zhian Bao[3], Zongsuo Liang[1, 4, *]

***Correspondence:** Zongsuo Liang **email:**liangzs@ms.iswc.ac.cn

1. Supplementary Data

1.1 The information of pre-experiment

There were 6 concentration gradients (0, 0.05, 0.2, 0.5, 1 and 2 mM) for pre-experiments. These concentration gradients could substantially represent lower to higher concentrations of Cr treatment in various plants [1-13] (Table S5). Cr at 1 mM concentration showed a little damage to plant growth and 2 mM Cr imposed a significant damage to plant growth. The purpose of our manuscript is to study Cr accumulation and transportation mechanisms in *Coptis chinensis* Franch.. To allow for study accumulation and transportation mechanisms of heavy metal in plants, it is important to understand the localization and speciation that under the excess heavy metal accumulation [14]. Therefore, we selected the high concentration (0.5mM) of Cr treatment, without destroying the normal growth of plants, to study the localization and speciation of Cr in *C. chinensis* Franch..

After we selected the treatment concentration, we also investigated the treatment time in the pre-experiment. We selected 0 h, 24 h, 5 d, 10 d, and 20 d to measure the Cr concentrations in the plants (Table S6). Table S6 shows that Cr concentrations in plants continued to increase until 10 d, but slowly between 10 d and 20 d, with little difference in Cr concentrations. Therefore, we selected 10 d as a treatment time. Additionally, there are two common and stable valence states of Cr in natural, i.e., Cr(III) and Cr(VI). Therefore, beside 10 d, we also select 24 h as another treatment time to study the speciation changes of Cr in *C. chinensis* Franch. with the short-term and long-term Cr treatment.

1.2 ICP-MS methods

1.2.1 Microwave digestion

For ICP-MS analysis, 0.5 g sample was accurately weighted into a Teflon digestion vessel. Next, 5ml concentrated HNO_3 and 1ml H_2O_2 (30%) were added and allowed to standing overnight. The vessels were closed and placed on the rotating turntable of the microwave oven (Mars 5, CEM, USA) and digested at 120°C for holding 5min, 160°C for holding 5min, and 180°C for holding 25min, with 15min ramp to temperature, at maximum power of 1,000 W, for 30min, and 0 W for 15min for cooling. Each sample was prepared in triplicate. After cooling, the solutions were evaporated to a small volume. Then the concentrated solutions were transferred to 50ml volumetric flasks, and 0.5ml HNO_3 was added before adjusting the final volume to 50ml with high purity deionized water. Sc was added to a final concentration of 100μg/L for its evaluation as internal standard. Blank digestion was carried out in the same way.

1.2.2 ICP-MS analysis

The ion lens settings, nebulizer gas flow rate and torch position of the ICP-MS were optimized in order to maximized the ion signals of the elements studied while reducing the background to a minimum. The argon gas utilized was of spectral purity (99.9998%). The sensitivity, stability, oxide levels, double charged ions, and resolution of the instrument were tested to achieve the requirements of determination. The instrumental settings and operative conditions were presented in Table S7.

1.2.3 Method validation

Internal standard solution with a concentration of 100μg/L Sc was used to correct changes in the sample uptake rate and plasma conditions for the ICP-MS measurements. The calibration equation was Y=3710.8X+1144.1 (R2=0.9998) for Sc. Certified Standard reference material (SRM) NIST 1547 Peach Leaves (National Institute of Standards and Technology, NIST, Gaithersburg, MD, USA) was used to assess the accuracy and the precision of the method. The measured concentration of Cr in Peach Leaves was 0.99 ± 0.02μg/g, while the certified concentration is 1μg/g.

1.3 LA-ICP-MS methods

1.3.1 Sample preparation

At harvesting, the roots and rhizomes were soaked in 20 mM Na_2-EDTA for 15 min to desorb surface-adsorbed Cr and then washed with deionized water. Sections (300-μm thick) for elemental microscopic imaging were prepared using a cryotome (Leica; CM3050S, Germany) at –20 ℃ and placed on tape prior to LA-ICP-MS analysis.

1.3.2 Standards compounds preparation and quantification procedure

Standard reference material NIST SRM 1547 Peach Leaves was applied for synthetic laboratory standard calibration. Preparation of a series of standard reference materials was according to Bei Wu et al. [15,16]. There were eight concentration gradients containing a mixture of elements of interest (Cu, Mn, P, Ca and Cr). The concentrations from 0.05 to 5 mg L^{-1} for Cu, 0.5 to 50 mg L^{-1} for Mn, 10 to 1,000 mg L^{-1} for P, 20 to 2,000 mg L^{-1} for Ca and from 1 to 100 mg L^{-1} for Cr were prepared by diluting the mixture of standard stock solution, and then added to the standard reference material. After absorption for 48h, the materials were dried, ground in an agate mortar, sieved to get particles smaller than 75μm and then pressed under 8 atm.

The prepared laboratory standards were scanned together with the samples and analyzed by LA-ICP-MS. The intensities of $^{63}Cu^+$, $^{55}Mn^+$, $^{31}P^+$, $^{43}Ca^+$ and $^{53}Cr^+$ were measured. The series of standards were scanned first, and then the tissue section was scanned line by line with a focused laser beam. We generated a series of two-dimensional elemental distributions profiles for C. chinensis Franch. petioles by scanning cross-sections line-by-line using a focused laser beam and analyzing them with mass spectrometry.

1.4 Samples and reference compounds preparation for XANES analysis

Roots and rhizomes were soaked in 20 mM Na_2–EDTA for 15 min to desorb putatively surface-adsorbed Cr. Then roots, rhizomes and leaves were immersed in liquid nitrogen until frozen and the placed in a vacuum freeze-dry System (GOLD-SIM, FD5-10T, USA). After lyophilization, the samples were ground and packed into 1.0 mm sample plates with 3M tape windows for analysis on beamline 1W1B at the Beijing Synchrotron Radiation Facility (BSRF).

There were ten Cr reference compounds, containing $K_2Cr_2O_7$ (solid), $Na_2CrO_4 \cdot 4H_2O$ (solid), $CrO_3 \cdot 6H_2O$ (solid), Cr_2O_3 (solid), Cr(III)-oxalate (solid), Cr(III)-phosphate ($CrPO_4 \cdot 4H_2O$) (solid), Cr(III)-acetate (solid), Cr foil, Cr(III)-histidine (solution) and Cr(III)-cysteine (solution). Solid reference compounds were purchased from Aladdin (Aladdin Industrial Corporation, California, USA) besides Cr(III)-oxalate, which was purchased from Sinopharm Chemical Reagent Beijing Co., Ltd (Shanghai, China). Cr(III)-histidine was synthesized according to Levina et al.[17]:the solution of $Cr(NO_3)_3 \cdot 9H_2O$ (Aladdin Industrial Corporation, USA) (5 mM in 15 ml of deionized water) was added dropwise with heating (79°C) and stirring to a solution of 25 mM L-histidine (CAS number 71-00-1) in 35 ml of deionized water. After the 1 hour reaction at 79°C, the pH was adjusted to 6.0 using 1M NaOH. Cr(III)-cysteine was prepared according to De Meester et al.[18]: 0.2 M 25 ml $Cr(NO_3)_3 \cdot 9H_2O$ was mixed with 1 M 25 ml L-cysteine (CAS number 52-90-4). After boiling the resulting solution for a few minutes, adjusted the pH to 6.0 using solid NaOH.

2. Supplementary Figures and Tables

Table S1. Cr concentration (mg kg^{-1} dry weight) in the coptidis rhizoma collected from three different location in Zhenping County, Ankang City, Shaanxi Province.

Location	Cr (mg kg^{-1})
Huaping town	2.48 ± 0.01
Xiaoshuhe town	3.11 ± 0.04
Zhongbao town	7.90 ± 0.21

Mean ± standard deviation (replicates = 3)

Table S2. Subcellular concentrations of Cr (mg kg^{-1} fresh weight) in different tissues of *C. chinensis* Franch..

Tissues	Treatments	Cr concentration (mg kg^{-1})			
		Cell wall	Organelle	Soluble fraction	Total
leaf	Cr0h-CK	0.237 ±0.011	0.087 ±0.004	0.055 ±0.002	0.379 ±0.017
	Cr0h	0.237 ±0.011	0.087 ±0.004	0.055 ±0.002	0.379 ±0.017
	Cr24h-CK	0.228 ±0.009	0.092 ±0.003	0.056 ±0.001	0.376 ±0.013
	Cr24h	0.513 ±0.022	0.075 ±0.001	0.141 ±0.006	0.729 ±0.029
	Cr10d-CK	0.253 ±0.012	0.098 ±0.001	0.068 ±0.001	0.419 ±0.014
	Cr10d	8.456 ±0.354	0.194 ±0.006	1.937 ±0.088	10.587 ±0.448
Rhizome	Cr0h-CK	1.007 ±0.037	0.077 ±0.001	0.067 ±0.001	1.151 ±0.039
	Cr0h	1.007 ±0.037	0.077 ±0.001	0.067 ±0.001	1.151 ±0.039
	Cr24h-CK	1.056 ±0.041	0.079 ±0.003	0.071 ±0.001	1.206 ±0.045
	Cr24h	2.371 ±0.056	0.137 ±0.004	0.552 ±0.022	3.060 ±0.082
	Cr10d-CK	1.041 ±0.032	0.079 ±0.002	0.071 ±0.003	1.191 ±0.037
	Cr10d	29.057 ±0.635	1.151 ±0.029	3.921 ±0.143	34.129 ±0.807
Root	Cr0h-CK	1.330 ±0.029	0.300 ±0.008	0.210 ±0.01	1.840 ±0.047
	Cr0h	1.330 ±0.029	0.300 ±0.008	0.210 ±0.01	1.840 ±0.047
	Cr24h-CK	1.322 ±0.024	0.292 ±0.006	0.196 ±0.009	1.810 ±0.039
	Cr24h	52.65 ±1.398	4.451 ±0.164	18.852 ±0.792	75.953 ±2.354
	Cr10d-CK	1.382 ±0.036	0.294 ±0.007	0.214 ±0.006	1.890 ±0.049
	Cr10d	218.985 ±5.897	11.632 ±0.439	50.566 ±2.033	281.183 ±8.369

Mean ±standard deviation (replicates = 3). The Cr concentration of each subcellular fraction is equal to the Cr content in each subcellular fraction divided by the tissues weight.

Table S3. Proportions of particular chromium species in *C. chinensis* Franch. samples as assessed by Cr Kα XANES linear combination fit (LCF) analysis.

	Cr0h			Cr 24h			Cr 10d		
	Leaves	Rhizomes	Roots	Leaves	Rhizomes	Roots	Leaves	Rhizomes	Roots
$CrPO_4$		14.1 ±3.0				32.7 ±2	51.6 ±0.5	43.5 ±2.2	41.3 ±0.4
$Cr(Ac)_3$		39.0 ±3.0		33.5 ±0.3	44.4 ±0.3				
Cr-Oxalate			24.6 ±4.1		15.7 ±0.2				
Cr-Histidine	3.5 ±0.1		8.2 ±4.3			34.8 ±2	29.2 ±0.4	31.8 ±2.2	22.3 ±0.3
Cr-Cysteine	28.8 ±0.3			3.6 ±0.1					
Cr_2O_3			13.4 ±3.3						
Cr foil	72.9 ±0.3	50.5 ±0.6	58.6 ±1.2	69.7 ±0.3	43.2 ±0.4		10.1 ±0.2		
CrO_3							10.7 ±0.2	23.0 ±1	35.5 ±0.3
$K_2Cr_2O_7$						27.0 ±5.9			
Na_2CrO_4						4.0 ±0.7			
sum	105.2	103.6	104.8	106.8	103.3	98.5	101.6	98.3	99.1
Residual	0.000846	0.000212	0.000475	0.001418	0.000310	0.000143	0.000119	0.000128	0.000263
Chi-square	0.06851	0.01835	0.04260	0.01913	0.02816	0.01187	0.01114	0.01117	0.02190

Table S4. Proportions of Cr in different subcellular fractions of leaf, rhizome, and root in *C. chinensis* Franch. at different treatment time.

Tissues	Subcellular fraction	Proportion (%)					
		Cr0h-CK	Cr0h	Cr24h-CK	Cr24h	Cr10d-CK	Cr10d
Leaf							
	Cell wall	62.6 ±0.8^{a}	62.6 ±0.8^{a}	60.5 ±1.2^{a}	70.2 ±1.3^{a}	60.4 ±0.9^{a}	79.9 ±0.7^{a}
	Organelle	22.9 ±0.7^{b}	22.9 ±0.7^{b}	24.5 ±0.8^{b}	10.2 ±0.3^{c}	23.4 ±0.6^{b}	1.8 ±0.01^{c}
	Soluble fraction	14.6 ±0.1^{c}	14.6 ±0.1^{c}	15.0 ±0.4^{c}	19.2 ±0.7^{b}	16.1 ±0.4^{c}	18.3 ±0.7^{b}
Rhizome							
	Cell wall	87.5 ±0.3^{a}	87.5 ±0.3^{a}	87.5±0.2^{a}	77.5 ±0.4^{a}	87.4 ±0.3^{a}	85.1 ±0.3^{a}
	Organelle	6.7 ±0.2^{b}	6.7 ±0.2^{b}	6.6 ±0.04^{b}	4.5 ±0.1^{c}	6.6 ±0.2^{b}	3.4 ±0.01^{c}
	Soluble fraction	5.8 ±0.1^{c}	5.8 ±0.1^{c}	5.9 ±0.1^{c}	18.0 ±0.3^{b}	6.0 ±0.2^{c}	11.5 ±0.3^{b}
Root							
	Cell wall	72.3 ±0.5^{a}	72.3 ±0.5^{a}	73.1 ±0.6^{a}	69.3 ±0.3^{a}	73.1 ±0.8^{a}	77.9 ±0.3^{a}
	Organelle	16.3 ±0.3^{b}	16.3 ±0.3^{b}	16.1 ±0.1^{b}	5.9 ±0.04^{c}	15.5 ±0.4^{b}	4.1 ±0.05^{c}
	Soluble fraction	11.4 ±0.3^{c}	11.4 ±0.3^{c}	10.6 ±0.3^{c}	24.8 ±0.3^{b}	11.3 ±0.5^{c}	18.0 ±0.3^{b}

Mean ± standard deviation (replicates = 3). The Cr proportion of each subcellular fraction is equal to the Cr content in each subcellular fraction divided by the total Cr content in tissues. Values with different letters in the same column indicate a significant difference at $p < 0.05$.

Table S5. Experimental concentrations and period of Cr in various plants.

Plant	Chromium exposure Cr(VI)	Experiment period	Reference
Subterranean Clover	0, 0.04, and 1.6 mM	4 and 21 days	Howe et al., 2003[1]
Wheat	0, 0.25 and 0.5 mM	7 days	Adrees et al., 2015[2]
Cauliflower	0.5 mM	30 days	Chatterjee and Chatterjee, 2000[3]
Gynura pseudochina (L.) DC.	100 mg L^{-1}	14 days	Mongkhonsin et al., 2011[4]
Prosopis laevigata	0.0, 0.5, 1.0, 2.0, 3.4 mM	50 days	Buendíagonzález et al., 2010[5]
Callitriche cophocarpa Sendtn.	50, 100, 400 or 700 μM	5, 10 or 24 days	Augustynowicz et al., 2010[6]
Raphanus sativus L.	2.0, 3.5, 5.0, 6.5 and 8.0 mM	0, 1, 3, 6 and 12 h	Sayantan and Shardendu, 2013[7]
Datura innoxia	0, 0.05, 0.1, 0.2, 0.5, 1 and 2 mM	21 days	Vernay et al., 2008[8]
Genipa americana L.	0, 15 and 30 mg L^{-1}	15 days	Santana et al., 2012[9]
Mesquite	0, 20, 40, 75, and 125 mg L^{-1}	15 days	Arias et al., 2010[10]
Halimione portulacoides	0, 15 and 30 mg L^{-1}	0 and 7 days	Duarte et al., 2012[11]
Alternanthera philoxeroides	0, 25, 50, 100, 150 mg L^{-1}	12 days	Vajravel and Saravanan, 2013[12]
Miscanthus sinensis	0, 50, 100, 200, 300, 500, 750 and 1000 μM	3 days	Sharmin et al., 2012[13]

Table S6. Concentrations of Cr (mg kg^{-1} fresh weight) in plant of *C. chinensis* Franch. at different treatment time in pre-experiment.

Treatment time	Cr (mg kg^{-1})
0 h	3.498 ± 0.080^{d}
24 h	79.166 ± 2.097^{c}
5 d	183.050 ± 6.380^{b}
10 d	329.154 ± 8.957^{a}
20 d	335.253 ± 4.653^{a}

Mean ± standard deviation (replicates = 3). Values with different letters in the same column indicate a significant difference at $p < 0.05$.

Table S7. ICP-MS operating conditions and measurement parameters.

Spectrometer	Varian ICP-820MS (Varian,USA)
Nebulizer	Meinhard
Spray chamber	Scott
PF power(KW)	1.4
Ar gas flow rate (L min^{-1})	28
Plasma gas flow rate (L min^{-1})	18
Auxiliary gas flow rate (L min^{-1})	1.8
Nebulizer	0.85
Lens voltage(V)	6.25
Torch horizontal alignment (mm)	0.8
Torch vertical alignment (mm)	0.5
Scanning mode	Peak-hopping
Resolution (amu)	0.8
Dwell time (ms)	10
Sampling depth (mm)	7.5
Sample uptake rate (ml min^{-1})	0.15

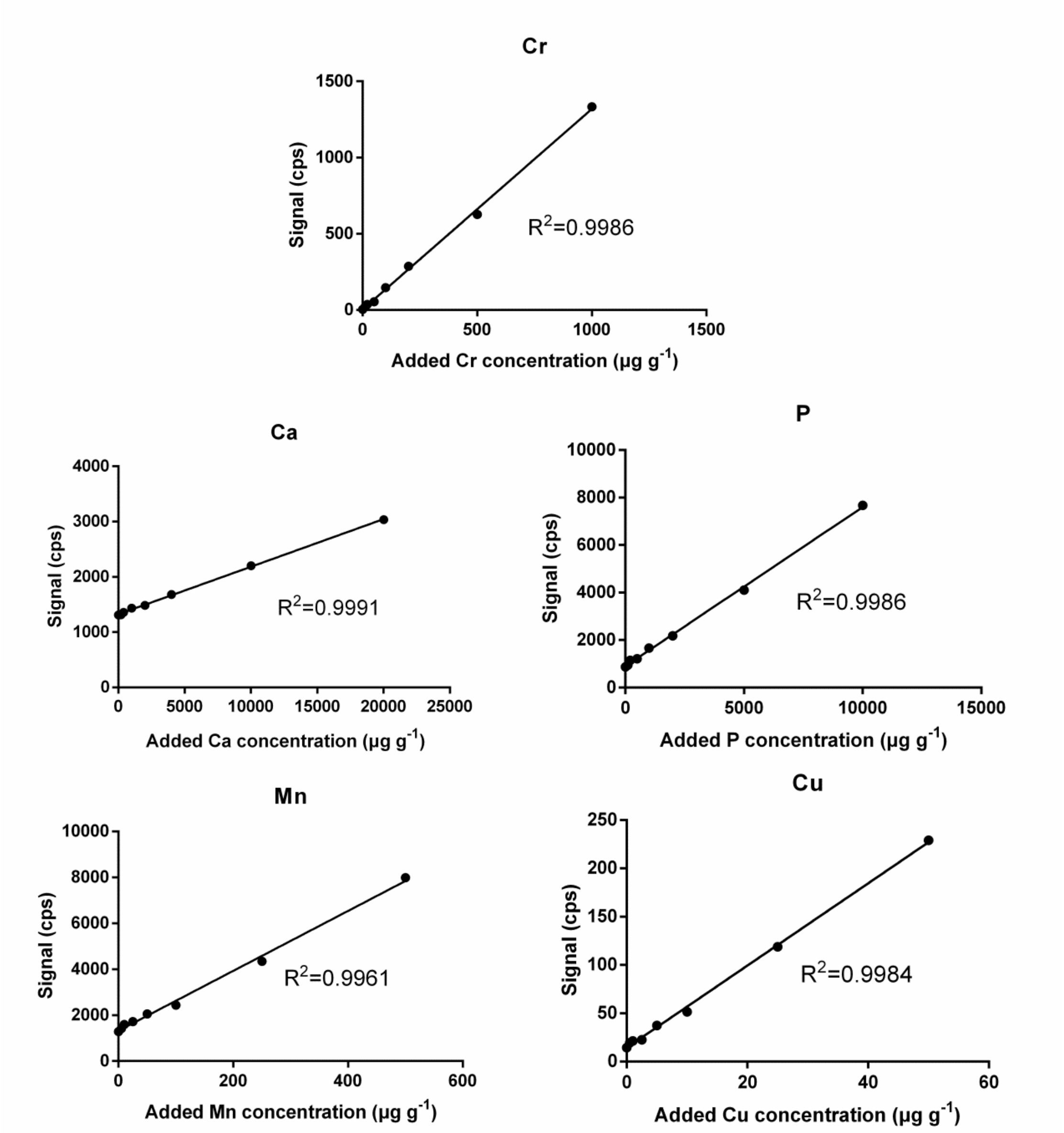

Figure S1. Calibration curves measured by LA-ICP-MS using prepared laboratory standards NIST SRM 1547 Peach Leaves doped with standard solutions of the analytes of known concentration.

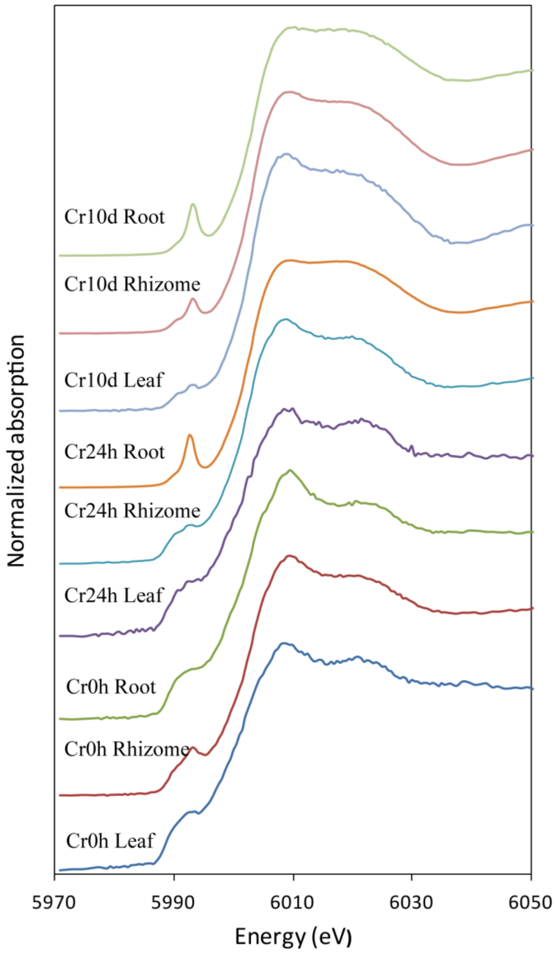

Figure S2. XANES data of *C. chinensis* Franch. roots, rhizomes and leaves of the Cr0h, Cr 24h and Cr10d groups.

References

1 Howe, J. A., Loeppert, R. H., Derose, V. J., Hunter, D. B. & Bertsch, P. M. Localization and speciation of chromium in subterranean clover using XRF, XANES, and EPR spectroscopy. *Environmental Science & Technology* **37**, 4091-4097 (2003).

2 Adrees, M. *et al.* Mannitol alleviates chromium toxicity in wheat plants in relation to growth, yield, stimulation of anti-oxidative enzymes, oxidative stress and Cr uptake in sand and soil media. *Ecotoxicology & Environmental Safety* **122**, 1 (2015).

3 Chatterjee, J. & Chatterjee, C. Phytotoxicity of cobalt, chromium and copper in cauliflower. *Environmental pollution* **109**, 69-74 (2000).

4 Mongkhonsin, B., Nakbanpote, W., Nakai, I., Hokura, A. & Jearanaikoon, N. Distribution and speciation of chromium accumulated in Gynura pseudochina (L.) DC. *Environmental & Experimental Botany* **74**, 56-64 (2011).

5 Buendíagonzález, L., Orozcovillafuerte, J., Cruzsosa, F., Barreradíaz, C. E. & Vernoncarter, E. J. Prosopis laevigata a potential chromium (VI) and cadmium (II) hyperaccumulator desert plant. *Bioresour Technol* **101**, 5862-5867 (2010).

6 Augustynowicz, J. *et al.* Chromium(VI) bioremediation by aquatic macrophyte Callitriche cophocarpa Sendtn. *Chemosphere* **79**, 1077-1083 (2010).

7 Sayantan, D. & Shardendu. Amendment in phosphorus levels moderate the chromium toxicity in Raphanus sativus L. as assayed by antioxidant enzymes activities. *Ecotoxicology & Environmental Safety* **95**, 161-170 (2013).

8 Vernay, P. *et al.* Effect of chromium species on phytochemical and physiological parameters in Datura innoxia. *Chemosphere* **72**, 763-771 (2008).

9 Santana, K. B. *et al.* Physiological analyses of Genipa americana L. reveals a tree with ability as phytostabilizer and rhizofilterer of chromium ions for phytoremediation of polluted watersheds. *Environmental & Experimental Botany* **80**, 35-42 (2012).

10 Arias, J. A. *et al.* Effects of Glomus deserticola inoculation on Prosopis: enhancing chromium and lead uptake and translocation as confirmed by X-ray mapping, ICP-OES and TEM techniques. *Environmental & Experimental Botany* **68**, 139-148 (2010).

11 Duarte, B., Silva, V. & Caçador, I. Hexavalent chromium reduction, uptake and oxidative biomarkers in Halimione portulacoides. *Ecotoxicology & Environmental Safety* **83**, 1-7 (2012).

12 Vajravel, S. & Saravanan, P. Accumulation of chromium and its effects on physiological and biochemical parameters of Alternanthera philoxeroides seedlings. *Journal of Pharmacy Research* **7**, 633-639 (2013).

13 Sharmin, S. A. *et al.* Chromium-induced physiological and proteomic alterations in roots of Miscanthus sinensis. *Plant Science An International Journal of Experimental Plant Biology* **187**, 113 (2012).

14 Li, L. Z., Tu, C., Peijnenburg, W. J. & Luo, Y. M. Characteristics of cadmium uptake and membrane transport in roots of intact wheat (Triticum aestivum L.) seedlings. *Environmental pollution* **221**, 351-358 (2017).

15 Wu, B., Zoriy, M., Chen, Y. & Becker, J. S. Imaging of nutrient elements in the leaves of Elsholtzia splendens by laser ablation inductively coupled plasma mass spectrometry (LA-ICP-MS). *Talanta* **78**, 132-137, doi:10.1016/j.talanta.2008.10.061 (2009).

16 Wu, B., Chen, Y. & Becker, J. S. Study of essential element accumulation in the leaves of a Cu-tolerant plant Elsholtzia splendens after Cu treatment by imaging laser ablation

inductively coupled plasma mass spectrometry (LA-ICP-MS). *Analytica Chimica Acta* **633**, 165-172, doi:10.1016/j.aca.2008.11.052 (2009).

17 Levina, A., Harris, H. H. & Lay, P. A. X-ray absorption and EPR spectroscopic studies of the biotransformations of chromium(VI) in mammalian cells. Is chromodulin an artifact of isolation methods? *Journal of the American Chemical Society* **129**, 1065 (2007).

18 Meester, P. D., Hodgson, D. J., Freeman, H. C. & Moore, C. J. ChemInform Abstract: TRIDENTATE COORDINATION BY THE L-CYSTEINE DIANION. CRYSTAL AND MOLECULAR STRUCTURE OF SODIUM BIS(L-CYSTEINATO)CHROMATE(III) DIHYDRATE. *Inorganic Chemistry* **8**, 1494-1498 (1977).
